# MaxEnt with remote sensing for tea plantation suitability under climate change

**DOI:** 10.1016/j.isci.2026.115887

**Published:** 2026-04-27

**Authors:** Shijie Wu, Pengfei Tian, Xiaochen Zhu, Hengxin Dong, Shiwen Liu

**Affiliations:** 1 Jiangsu Provincial University Key Laboratory of Agricultural and Ecological Meteorology, Nanjing University of Information Science and Technology, Nanjing 210044, Jiangsu, China; 2 Nanjing Meteorological Bureau, Nanjing 210019, China; 3 Shandong Meteorological Bureau, Jinan 250031, China

**Keywords:** ecology, remote sensing, agricultural science

## Abstract

Premium tea cultivation is highly vulnerable to climate change, yet its future suitability remains insufficiently understood. In this study, we integrated spatially de-biased tea occurrence records derived from Gaofen-6 imagery and a U-Net deep learning framework with the MaxEnt model to project tea suitability in Nanping, Southeastern China, under multiple CMIP6 climate scenarios from 2021 to 2080. Random forest was used to cross-check model robustness. The results show that precipitation seasonality and precipitation of the wettest quarter are the main climatic drivers of tea suitability, and future warming is likely to shift highly suitable areas southward while increasing spatial fragmentation under high-emission scenarios. These findings provide a transferable framework for evaluating climate-sensitive high-value crops and support more adaptive agricultural planning under global environmental change.

## Highlights

• Integrates remote sensing, U-Net mapping and MaxEnt for high-resolution maps
• Strong predictive performance for tea suitability (AUC = 0.992, TSS = 0.948, and CBI = 0.976)
• Identifies bio16 and bio15 as dominant climatic predictors
• Identifies southward habitat infiltration driven by relaxed thermal constraints

## Graphical abstract

**Figure:**
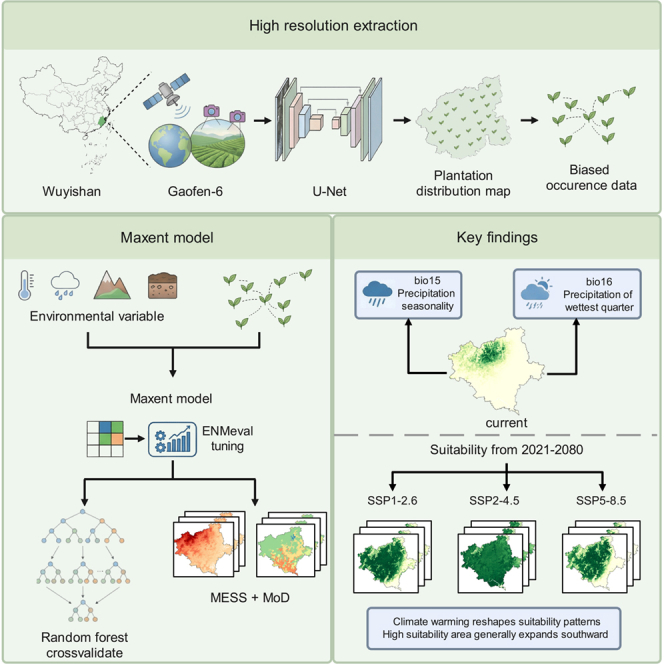

## Introduction

Tea (*Camellia sinensis*) is a climate-sensitive perennial cash crop and one of the three most consumed beverages worldwide. Its shoot growth, yield, and biochemical quality respond strongly to temperature and precipitation variability and extremes, rendering tea production particularly exposed to climate risk.^1^^,^^2^ China remains the leading global producer, with an output of 3.74 million tons in 2024—over half of global production—while also serving as a major exporter (374,100 tons; USD 1.419 billion).^3^^,^^4^^,^^5^ Fujian Province, especially the Wuyi Mountains, is a key production area renowned for high-quality teas and distinctive terroir.^6^^,^^7^ Of particular note is the iconic Da Hong Pao brand; with a brand valuation exceeding USD 10 billion, it exemplifies the deep historical legacy of imperial tribute tea and the substantial economic premium of the region’s industry.^8^ Recent rural revitalization policies have stimulated local production, generating employment and improving farmers’ incomes. Yet, the rapid expansion of monoculture plantations at the expense of forests and other natural land covers has led to biodiversity loss, soil degradation, erosion, and heightened pest pressure.^9^^,^^10^^,^^11^

Superimposed on existing land-use pressures, climate change exacerbates the risks of drought and extreme precipitation. These stressors threaten tea yield and quality by disrupting phenological traits, compromising soil health, and facilitating the spread of pests and diseases.^12^^,^^13^ Crucially, the impact of climate change on tea cultivation patterns transcends simple sensitivity responses; it involves complex dynamic processes including the contraction, expansion, and migration of suitable habitats.^14^ The underlying mechanism driving these shifts resides in the profound coupling of climatic factors and socioeconomic decision-making. Climate change modulates the synthesis and accumulation ratios of secondary metabolites, directly determining the flavor profile and subsequent market premiums of tea.^13^^,^^15^ Research indicates that declines in functional quality induced by climate extremes significantly suppress grower preferences and lead to substantial reductions in household income. This shift in economic incentives drives spatial adjustments in cultivation, characterized by the abandonment of low-quality legacy zones in favor of new suitable areas at higher altitudes or latitudes.^15^^,^^16^ Currently, large-scale studies on shifting tea suitability exhibit significant discrepancies, particularly regarding projections for China. Some models predict an average reduction of approximately 4.7% in China’s optimal suitability areas by 2050, with a continuing downward trend.^17^ Conversely, other scholars suggest that driven by expansion in mid-to-high latitudes, China’s suitable tea-growing area may actually increase by 2.7%–31.5%, accompanied by a distinct northward shift.^18^ Such inconsistencies often stem from the coarse granularity of global or national-scale models, which struggle to capture the fine-grained influences of local topography and regional microclimates. Consequently, when downscaling the research focus to specific regions like Nanping, the evolutionary trajectory of tea cultivation suitability remains shrouded in high uncertainty. These interacting anthropogenic and climatic drivers threaten the long-term sustainability of tea production and rural livelihoods, underscoring the need for filling these knowledge gaps through regional assessments of suitability shifts and targeted adaptation strategies in core producing regions.

Remote sensing provides an essential pathway to map crop distributions and track land-cover change. Compared with field surveys, satellite observations offer superior spatial coverage, timeliness, and cost-effectiveness, enabling large-area, high-precision crop distribution databases^19^^,^^20^ and supporting continuous validation and near-real-time updates for spatial modeling.^21^ Early mapping relied on manual visual interpretation—labor-intensive and subjective^22^—followed by pixel-based classifiers focused on spectral features. Such approaches suffer from spectral confusion and neglect texture and context, limiting accuracy.^23^^,^^24^ Object-based image analysis alleviates salt-and-pepper noise by segmenting images into homogeneous objects and leveraging spectral, textural, shape, and contextual cues,^25^ but it still depends on expert-driven feature engineering.^26^^,^^27^ Deep learning—particularly convolutional neural networks —has transformed feature extraction through end-to-end training.^28^^,^^29^ Transitioning from patch-based classifiers to fully convolutional networks enabled efficient, pixel-level semantic segmentation.^30^ As a representative encoder-decoder architecture, U-Net uses skip connections to fuse high-resolution encoder features with decoder outputs, preserving spatial detail and improving boundary delineation and small-object detection capabilities well suited to complex tea-plantation patterns in high-resolution imagery.^31^^,^^32^^,^^33^

Species distribution models (SDMs) are widely used to quantify relationships between species occurrences and environmental gradients, supporting conservation planning, invasive species control, and ecological restoration amid accelerating climate change and biodiversity loss.^34^ MaxEnt, a leading presence-only SDMs grounded in the principle of maximum entropy, performs robustly with limited samples, captures nonlinear responses, and employs regularization to limit overfitting, yielding interpretable predictions.^34^^,^^35^^,^^36^^,^^37^^,^^38^ Remote sensing augments SDMs by providing high-resolution, spatially explicit predictors—such as vegetation indices, land surface temperature, and land cover—that complement coarse bioclimatic and topographic variables and enhance the characterization of complex planting landscapes.^39^^,^^40^^,^^41^^,^^42^^,^^43^^,^^44^ In recent years, remote sensing has emerged as a pivotal auxiliary tool for SDMs, though its application remains largely confined to providing environmental predictor layers.^45^^,^^46^ Driven by methodological advancements, researchers have begun exploring the direct conversion of remote-sensing classification results into occurrence records, aimed at mitigating the spatial coverage and timeliness constraints inherent in conventional field surveys.^47^^,^^48^ Despite extensive efforts to assess the impacts of climate change on tea suitability, several critical limitations persist regarding data precision, model integration, and regional application. First, concerning spatial resolution and feature characterization, many large-scale studies rely on low-to-medium resolution data or coarsened bioclimatic variables. In the Wuyi Mountain region, characterized by rugged topography and pronounced microclimatic gradients, such data fail to accurately identify small-scale plantation patches. These approaches are frequently compromised by mixed pixel effects, which obscure the precise delineation of plantation boundaries and actual cultivation patterns.^33^^,^^49^ Second, bottlenecks in identification accuracy and sampling bias continue to hinder traditional extraction and modeling workflows. While attempts have been made to couple remote sensing (RS) with SDMs, they predominantly depend on conventional machine learning or simplistic spectral indices. These methods often lack the precision required to resolve spectral confusion and complex landscape textures.^23^ Furthermore, traditional SDMs depend on opportunistic records from databases such as Global Biodiversity Information Facility (GBIF) or field surveys, leading to pronounced spatial sampling bias.^50^^,^^51^^,^^52^^,^^53^ Finally, current research rarely addresses the quantitative assessment of scientific variety introduction from core premium production hubs to potential expansion zones.^14^ Regional-scale studies that evaluate the feasibility of high-quality tea expansion based on environmental thresholds and cultivation viability remain significantly underdeveloped.^14^^,^^54^

**Figure 1:**
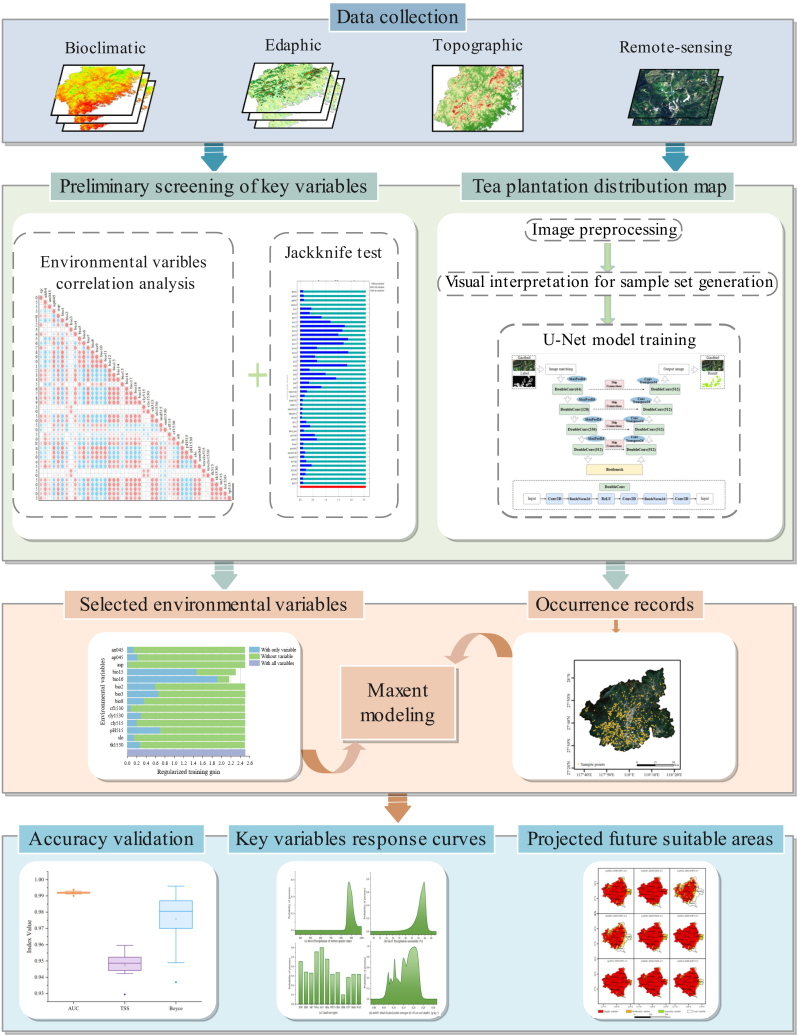
Framework of this study

To address these limitations, we developed a reproducible, highly versatile, and scalable integrated framework that couples high-resolution remote sensing interpretation with MaxEnt modeling to systematically quantify the evolutionary trends of tea cultivation suitability and introduction potential under climate change (Figure 1). Methodologically, the workflow first employs a U-Net semantic segmentation model to perform deep interpretation of Chinese Gaofen-6 imagery, effectively overcoming identification bottlenecks posed by complex terrain and fragmented landscapes, achieving high-precision extraction of fine-grained tea plantation distributions within core production zones. Subsequently, leveraging these exhaustive RS-derived results, we generated a high-fidelity, de-biased occurrence dataset through spatial thinning to mitigate sampling artifacts. This approach not only compensates for the scarcity of field survey data but also fundamentally eliminates intrinsic sampling bias. This framework provides robust spatial decision support for regional plantation layout optimization and the strategic expansion of premium varieties from core terroirs to broader administrative scales amid escalating climate risks.

## Results

### High-resolution tea plantation distribution map

**Figure 2:**
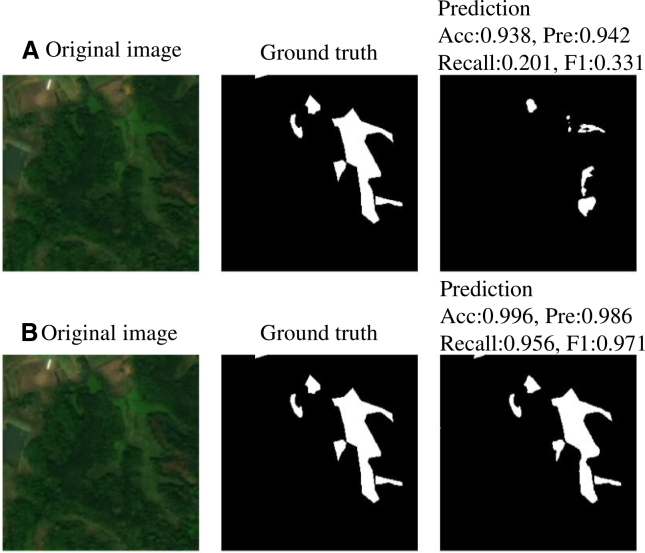
Comparison of model prediction performance at different training stages Comparison of original images, ground-truth labels, and model predictions at different training epochs: (A) Epoch 10; (B) Epoch 100.

**Figure 3:**
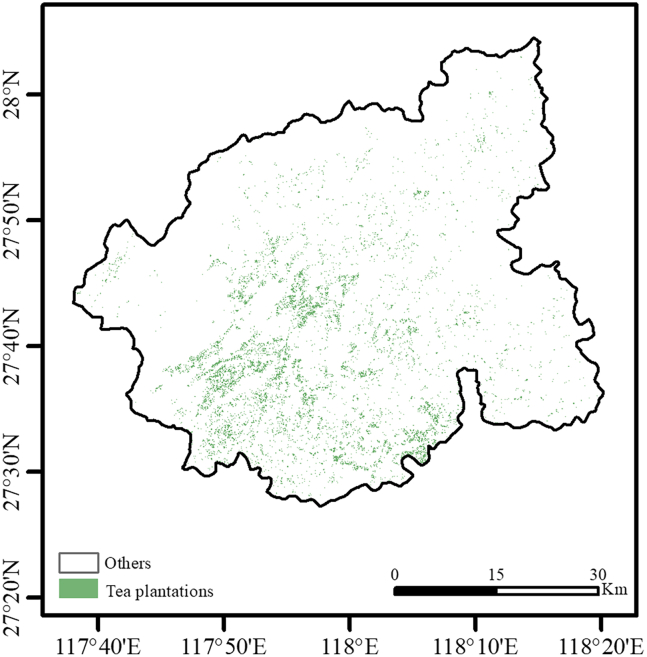
Spatial distribution of tea plantations in Wuyishan City The scale bar indicates 0–30 km.

Figure 2 illustrates the predictive evolution during U-Net training. At the initial stage (epoch 10), fragmented tea patches were partially identified, but they exhibited pronounced fragmentation and omission errors. This indicates that the model required a deeper acquisition of spatial-semantic features related to cultivation patterns. By epoch 100, enhanced patch continuity, refined boundaries, and a significant reduction in omissions demonstrated the model’s capacity to effectively learn multiscale spatial patterns and their coupling with spectral responses. The final classification results for Wuyishan City (Figure 3) yielded an accuracy of 0.993, a precision of 0.972, a recall of 0.935, and an F1 score of 0.953. The concurrent high precision and recall confirm the effective suppression of false positives while comprehensively capturing actual tea pixels. This is critical for MaxEnt modeling, as contiguous, low-fragmentation occurrence records enable more precise niche characterization, thereby enhancing model interpretability and stability.

Spatial consistency validation through township-level area statistics (Table 1) revealed a strong agreement with the 2021 statistical yearbooks (mean absolute error [MAE] = 1.51% and root mean square error [RMSE] = 2.45%). Specifically, minor underestimations were observed in core production zones such as Xingcun town (−2.83 percentage points [pps]) and Wuyi Subdistrict (−0.55 pp), while overestimations were noted in Xingtian town (+6.85 pp) and Yangzhuang township (+3.60 pp). These positive deviations may indicate post-statistical cultivation expansion or intensified management, highlighting the temporal sensitivity of remote sensing. The limited overestimations could potentially arise from spectral confusion between evergreen shrubs and young orchards. However, the minimal overall errors can be mitigated through threshold optimization, manual verification, or multitemporal analysis. The spatial consistency confirms the significant advantages of remote sensing-derived occurrence data for MaxEnt: comprehensive spatial coverage, high resolution, and enhanced contiguity substantially reduce traditional sampling costs. Furthermore, high-precision identification authentically reflects cultivation patterns, thereby improving operational utility for site selection and pilot implementation.

**Table 1:** Discrepancy analysis: GF-6 satellite vs. Fujian Statistical Yearbook (2021) for tea cultivation area in Wuyishan City

Area	GF-6 based (%)	Statistical yearbook (%)	Abs error (%)
Xingcun town	38.28	41.11	2.83
Xingtian town	23.36	16.51	−6.85
Wuyi subdistrict	18.31	18.86	0.55
Yangzhuang township	7.33	3.73	−3.60
Wutun township	2.71	2.00	−0.71
Shangmei township	2.44	2.29	−0.14
Chong’an subdistrict	2.40	2.33	−0.08
Wufu town	2.20	3.50	1.30
Langu township	1.96	2.30	0.34
Xinfeng subdistrict	0.43	0.60	0.17
Comprehensive farm	0.41	0.72	0.31
Wuyishan city tea farm	0.16	1.35	1.18

Data represent the percentage of tea cultivation area relative to the total area of each administrative unit. Absolute error is calculated as the difference between the Statistical Yearbook value and the GF-6 based value.

### MaxEnt performance and influential environmental variables

**Figure 4:**
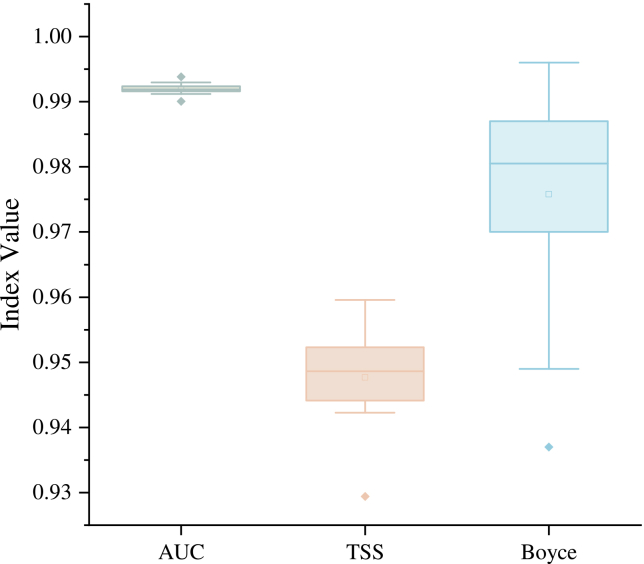
AUC, TSS, and CBI value of the MaxEnt model Data are represented as boxplots where the horizontal line within the box represents the median, the small square represents the mean, and the box boundaries indicate the 25th and 75th percentile. The whiskers extend to 1.5 times the interquartile range, and individual points represent outliers (*n* = 10 replicates).

**Figure 5:**
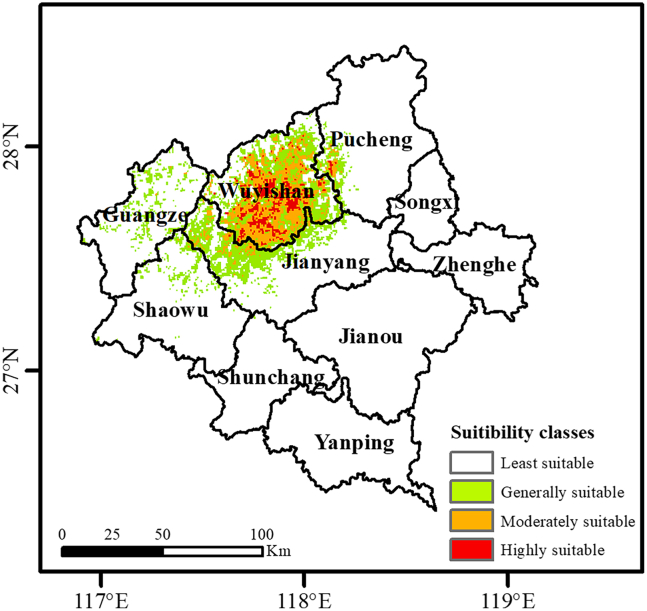
Current suitability classes map of *Camellia sinensis* based on MaxEnt model The scale bar indicates 0–100 km.

Based on ENMeval tuning, the MaxEnt model was constructed using a specific configuration of linear, quadratic, and hinge feature class (FC) combined with a regularization multiplier (RM) of 0.5. Evaluated through 4-fold cross-validation, the model demonstrated exceptional robustness and discriminatory power (Figure 4). Regarding the area under the receiver operating characteristic curve (AUC), the mean values for the training and testing sets were 0.994 and 0.992, respectively. The negligible difference of 0.002 between these values signifies high predictive accuracy while confirming the absence of significant overfitting. Furthermore, the mean true skill statistic (TSS) for the testing set reached 0.948; given that TSS integrates both sensitivity and specificity, this score further validates that the model’s classification precision far exceeds random expectations. Additionally, the continuous Boyce index (CBI) yielded a mean value of 0.976, indicating a high degree of consistency between the predicted suitability indices and the actual observed frequency of tea occurrences. Suitable cultivation areas were classified into four tiers using the maximum sensitivity-specificity threshold of 0.1984: 0–0.1984 (low suitability), 0.1984–0.3968 (moderate suitability), 0.3968–0.5952 (medium suitability), and 0.5952–1 (high suitability). Under current climatic scenarios (Figure 5), the suitability for tea cultivation in Nanping exhibits a distinct pattern characterized by central clustering and peripheral expansion. The core highly suitable areas are concentrated in Wuyishan City in the northwest, specifically across the central Wuyishan scenic area and its surrounding foothills traversed by the Wuyishan mountain range, which aligns with existing agricultural patterns. Furthermore, highly suitable zones extend in a punctate fashion toward the northern fringes of Pucheng County and select high-altitude hills within the Jianyang District. These high-suitability cores are predominantly encircled by moderately suitable zones, which radiate outward from Wuyishan City into the neighboring regions of Jianyang, Shaowu, and Pucheng.

**Figure 6:**
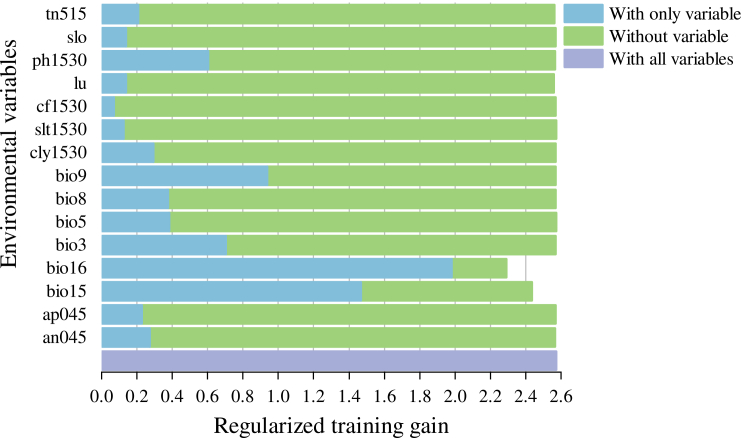
Results of jackknife evaluations of the environmental variables

The jackknife test results for environmental variables (Figure 6) identified several key factors: precipitation of the wettest quarter (bio16), precipitation seasonality (bio15), pH in the 5–15 cm soil layer (pH515), and mean temperature of driest quarter (bio9) and isothermality (bio3). These variables exhibited the highest regularization training gains, suggesting that they contain more ecologically informative signals. Furthermore, the variable contribution analysis (Table 2) confirmed that bio16 serves as the primary driver, with bio15 as a secondary contributor. Together, these two variables account for a cumulative contribution of 93% and a permutation importance of 90.8%, significantly surpassing other environmental factors. Furthermore, the ecological impacts of edaphic variables on tea plant distributions exhibited distinct vertical stratification across different soil horizons. The influence of soil nutrient availability was primarily concentrated in the deeper profiles, with alkali-hydrolyzable nitrogen (AN) and available phosphorus (AP) significantly mediating suitability within the 0–45 cm depth range. Soil acidity, a critical determinant for *Camellia sinensis*, was most influential at the intermediate layer (pH, 15–30 cm). Regarding soil physical properties, the influence of texture was multi-layered: total nitrogen (TN) showed a strong association with the topsoil (5–15 cm), while the structural components—comprising clay fraction, clay content, and silt content—exerted their primary effects at the 15–30 cm depth. This depth-dependent variation underscores the importance of subsoil characteristics in sustaining tea ecosystems and suggests that both nutrient-rich deep horizons and well-structured intermediate soil textures are essential for high-quality tea cultivation.

**Table 2:** Percentage contributions and permutation importance of the environment variables included in the MaxEnt models for *Camellia sinensis*

Variable	Percent contribution	Permutation importance
Bio16	69.1	48.5
Bio15	23.9	42.3
LU	3.2	0.7
AP045	0.8	0.2
AN045	0.8	0.2
PH1530	0.6	0.8
CF1530	0.5	0.2
TN515	0.3	1.8
Slo	0.2	0.2
Bio5	0.2	0.5
Bio8	0.1	2.1
Cly1530	0.1	0.3
Bio9	0.1	1.1
Bio3	0.1	0.9
Slt1530	0.1	0.2

**Figure 7:**
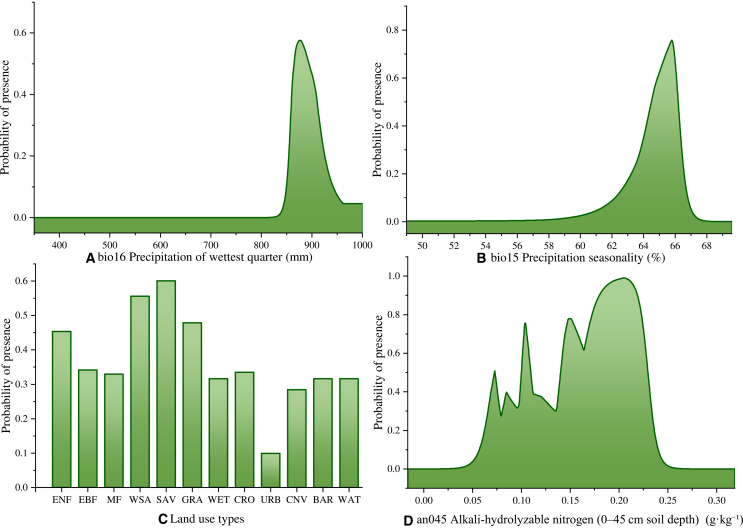
Response curves for important environmental variables in MaxEnt for *Camellia sinensis*

To elucidate the climatic determinants of potential cultivation areas for *Camellia sinensis* under contemporary conditions, response curves for four key environmental variables (Figure 7, cumulative contribution > 97%) reveal that the response to the bio16 exhibited a classic unimodal trajectory, reflecting the species’ sensitivity to hydrological extremes. The probability of presence rose sharply as precipitation increased, entering the generally suitable range between 860 and 909 mm, with a peak optimum at approximately 876 mm. Beyond 962 mm, suitability declined rapidly into the least suitable class, suggesting that while tea requires abundant moisture during the growing season, excessive rainfall may induce waterlogging stress or root hypoxia.^55^^,^^56^ The bio15 mirrored that of bio16, exhibiting a distinct unimodal pattern. Habitat suitability remains at the unsuitable level when bio15 values are ≤63%, followed by a sharp transition: environmental suitability surges to moderately suitable class at 64.2%, enters the highly suitable range at 64.8%, and reaches its maximum at 65.8%. Beyond this peak, suitability declines rapidly, returning to the unsuitable threshold once bio15 exceeds 66.8%. This sensitivity underscores that premium tea production in the region relies on a pronounced alternation between wet and dry seasons—a hallmark characteristic of the subtropical monsoon climate.^1^^,^^57^ Analysis of land-use types revealed distinct landscape preference patterns. Among the various land-cover categories evaluated, savannas (SAV) emerged as the most favorable habitat type and the only classification to attain a high suitability rating. Woody savannas (WSAs), grasslands (GRAs), and evergreen needleleaf forests (ENFs) followed, with suitability levels consistently falling within the moderately suitable range. All other land-use classes reached the generally suitable threshold, except for urban and built-up lands (URBs), which were deemed unsuitable due to their failure to meet minimum suitability requirements. This assessment aligns closely with empirical cultivation logic, effectively reflecting the functional exclusivity of built-up areas within agricultural spatial planning.^58^ The response profile of alkaline hydrolyzable nitrogen (AN045) reveals complex nonlinear dynamics characterized by pronounced multimodal fluctuations. Nitrogen initially acts as a stringent limiting factor; at concentrations below 0.041 g/kg, habitat suitability remains negligible. Within the 0.041–0.073 g/kg interval, suitability surges abruptly, surpassing the generally suitable class and moderately suitable class thresholds at 0.062 and 0.068 g/kg, respectively. Deviating from the typical smooth sigmoidal response curve, the high-suitability region exhibits a distinct serrated pattern with multiple local optima and troughs. Specifically, across the nitrogen-rich range (0.101–0.228 g/kg), the model identifies three prominent suitability peaks, with values consistently remaining above the generally suitable level. While these fluctuations reflect the model’s fine-scale sensitivity to local soil heterogeneity, the overarching trend confirms that once physiological nitrogen requirements are satisfied, the environment maintains a high probability of occurrence.

### Spatiotemporal dynamics of tea cultivation suitability

**Figure 8:**
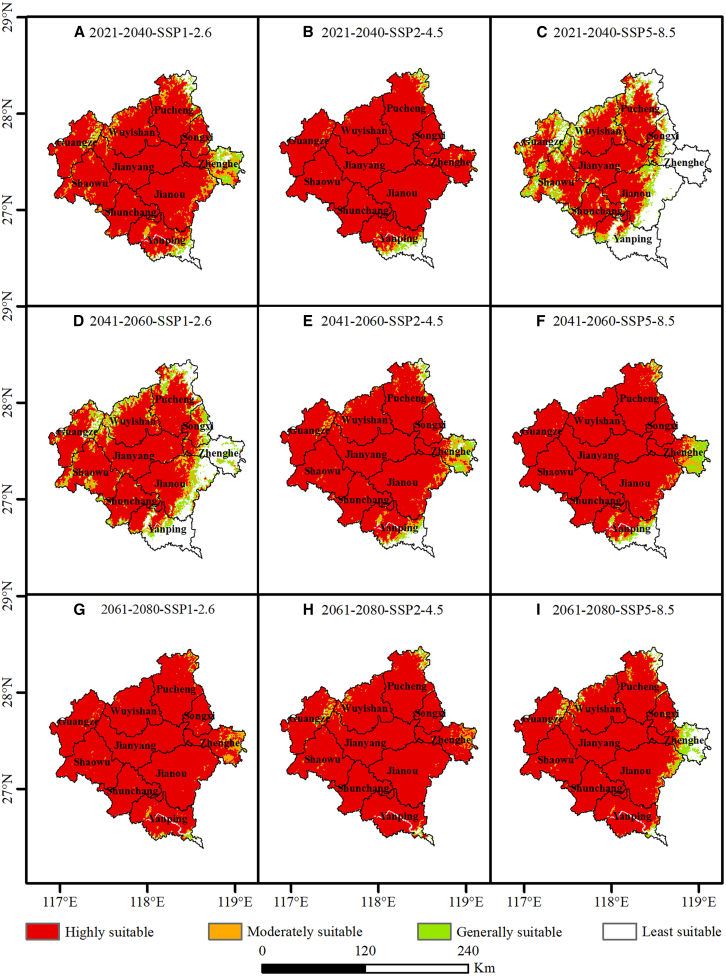
Suitability classes map of *Camellia sinensis* under future scenario based on MaxEnt model (A–C) 2021–2040 projections under SSP1-2.6, SSP2-4.5, and SSP5-8.5, respectively; (D–F) 2041–2060 projections under the three scenarios; (G–I) 2061–2080 projections under the three scenarios. The scale bar indicates 0–240 km.

At the regional scale, we evaluated the impacts of climate change on tea cultivation suitability across Nanping by analyzing spatiotemporal dynamics under three socioeconomic forcing pathway–representative concentration pathway (SSP-RCP) scenarios (SSP1-2.6, SSP2-4.5, and SSP5-8.5) for the near-term (2021–2040), mid-century (2041–2060), and late-century (2061–2080) periods (Figure 8). The result reveals significant spatiotemporal heterogeneity, where the magnitude of habitat shifts exhibits a non-linear amplification corresponding to both the progression of time and the escalation of radiative forcing intensities.

Under future climate scenarios, Nanping City exhibits immense potential for the introduction of premium tea cultivars, characterized by an extensive spatial expansion of highly suitable habitats. Relative to the baseline distribution, climate change appears to dismantle the existing environmental barriers of the Wuyi terroir by alleviating prevailing thermal constraints. By the 2040s, even under the sustainable SSP1-2.6 pathway, highly suitable areas are projected to surge to 22.12 × 10^3^ km^2^, encompassing 84.4% of Nanping’s total land area; by the 2080s, this expansion is expected to culminate at a peak extent of 25.20 × 10^3^ km^2^ (Table 3). This drastic spatial reorganization (Figure 8) underscores a pivotal trend: as regional warming progresses, the climatic profiles of southern and low-altitude zones traditionally deemed unsuitable—such as Shunchang, Yanping, and Jian’ou—will gradually converge with the current conditions of the Wuyishan core production zone. This shift creates a critical climatic window for the large-scale southward migration and introduction of elite Wuyi tea varieties.

**Table 3:** *Camellia sinensis* suitable cultivation area statistics across future scenarios in Nanping City, China

Period	Scenario	Highly suitable aera (×10^3^km^2^)	Moderately suitable aera (×10^3^km^2^)	Generally suitable aera (×10^3^km^2^)	Least suitable aera (×10^3^km^2^)
2021–2040	SSP1-2.6	22.12	2.04	1.21	8.20
	SSP2-4.5	24.74	0.49	0.38	5.78
	SSP5-8.5	13.36	2.72	2.83	72.77
2041–2060	SSP1-2.6	16.68	2.33	2.55	46.24
	SSP2-4.5	23.15	1.30	0.88	8.52
	SSP5-8.5	23.54	0.97	0.85	8.31
2061–2080	SSP1-2.6	25.20	0.73	0.17	0.84
	SSP2-4.5	25.08	0.68	0.29	1.29
	SSP5-8.5	23.30	0.88	0.91	10.91

Spatial dynamics further reveal a divergent pattern of lowland contraction versus highland expansion, primarily driven by topographic complexity. In terms of spatial heterogeneity and long-term stability, this introduction potential demonstrates high coherence across the Nanping region. Wuyishan’s status as a core habitat remains robust under all future scenarios, affirming its irreplaceable role as a primary germplasm repository, while peripheral regions such as Jianyang and Pucheng exhibit the most significant gains in habitat quality. Nevertheless, the formulation of introduction strategies must account for late-century climatic risks. Statistical data indicate a slight contraction in highly suitable areas under the long-term SSP5-8.5 scenario, accompanied by an increase in moderately suitable zones and signs of habitat fragmentation in southern low-elevation valleys, such as Yanping and Shunchang. This suggests that excessive warming driven by extreme greenhouse gas emissions may trigger negative feedback for low-altitude introduction environments by the end of the century.

Consequently, future strategic planning must account for scenario-specific uncertainties. Introduction planning should prioritize the 2040s–2060s window to leverage the climatic surplus, strategically dispersing Wuyishan’s premium germplasm to high-altitude zones in central and northern Nanping. The establishment of multi-site introduction trials across altitudinal gradients will be essential to ensure that the tea industry can achieve dynamic spatial optimization by tracking suitable ecological niches amid intensifying climatic volatility, ultimately securing high-quality production spaces at higher elevations.

### Evaluation of projection reliability under novel climates

To further validate the robustness of MaxEnt spatial predictions, we employed a random forest (RF) model for cross-validation. The RF model demonstrated highly reliable predictive performance, yielding an AUC of 0.947, a TSS of 0.792, and a CBI of 0.993. Although RF yielded slightly lower AUC and TSS values compared to MaxEnt, the exceptionally high CBI indicates strong agreement between predicted suitability and observed *Camellia sinensis* occurrence frequencies, confirming the robustness of its predictive capacity. Given the fundamental differences in algorithmic architecture and probability estimation between RF and MaxEnt, the MTSS thresholds varied; the RF model produced an MTSS value of 0.5429, notably higher than that of MaxEnt. Adopting this threshold within an equal-interval classification scheme, RF suitability predictions were categorized into four classes: least suitable (0–0.5429), generally suitable (0.5429–0.6953), moderately suitable (0.6953–0.8477), and highly suitable (0.8477–1). Spatial comparisons revealed that, despite differing classification thresholds, the suitability distribution identified by RF showed high consistency with MaxEnt outputs (Figure S1). Similarly, RF projections exhibited a pronounced aggregation of highly suitable areas centered on Wuyishan, extending toward Pucheng County and the hilly terrain of Jianyang District. This strong cross-algorithm spatial concordance indicates that the Wuyishan-centered suitability pattern is unlikely to represent an artifact of a specific modeling algorithm but rather reflects a robust, algorithm-independent ecological signal. These results reinforce the designation of Wuyishan as the core region for high-quality tea introduction and cultivation within Nanping.

**Figure 9:**
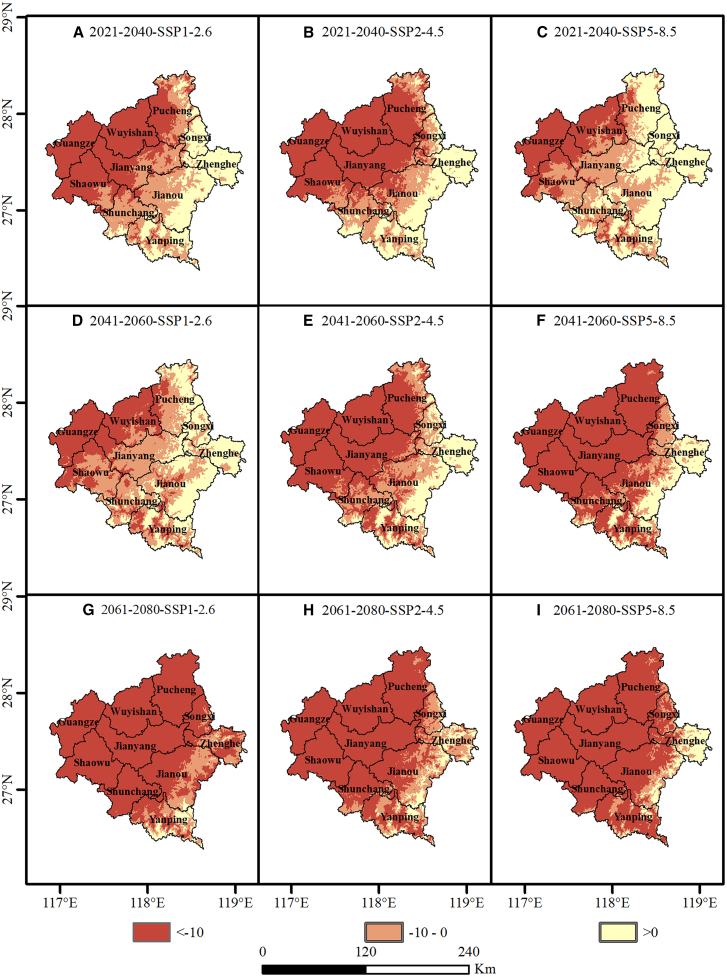
Spatial distribution of MESS scores under future climate scenarios (A–C) 2021–2040 projections under SSP1-2.6, SSP2-4.5, and SSP5-8.5, respectively; (D–F) 2041–2060 projections under the three scenarios; (G–I) 2061–2080 projections under the three scenarios. The scale bar indicates 0–240 km.

Complementing the algorithmic validation, we further evaluated the spatial reliability across the entire Nanping region using multivariate environmental similarity surface (MESS) and most dissimilar variable (MoD) analyses. MESS results revealed pronounced north-south spatial heterogeneity across all climate scenarios from 2021 to 2080 (Figure 9). Northern Nanping, including Wuyishan, Pucheng, and Guangze Counties, exhibited extensive strongly negative MESS values, predominantly below −10, indicating high extrapolation risk. Conversely, southern Nanping, including Yanping District, Shunchang County, and southern Jianyang District, maintained relatively high environmental similarity, with some areas exceeding MESS values of 0, thus remaining within a reliable interpolation domain. This pattern suggests that although training occurrence records were primarily derived from Wuyishan, the environmental relationships established during MaxEnt model construction based on baseline data covering the entire Nanping region indicate that northern production areas are likely to experience greater climatic novelty under future climate change. Localized MESS and MoD analyses focusing on the Wuyishan region provided in the Appendix (Figures S2 and S3) support this interpretation. These supplementary results demonstrate strong spatial continuity of high-risk extrapolation zones, indicating that the projected expansion of suitable areas across Nanping accompanies systematic shifts in the regional climate system.

**Figure 10:**
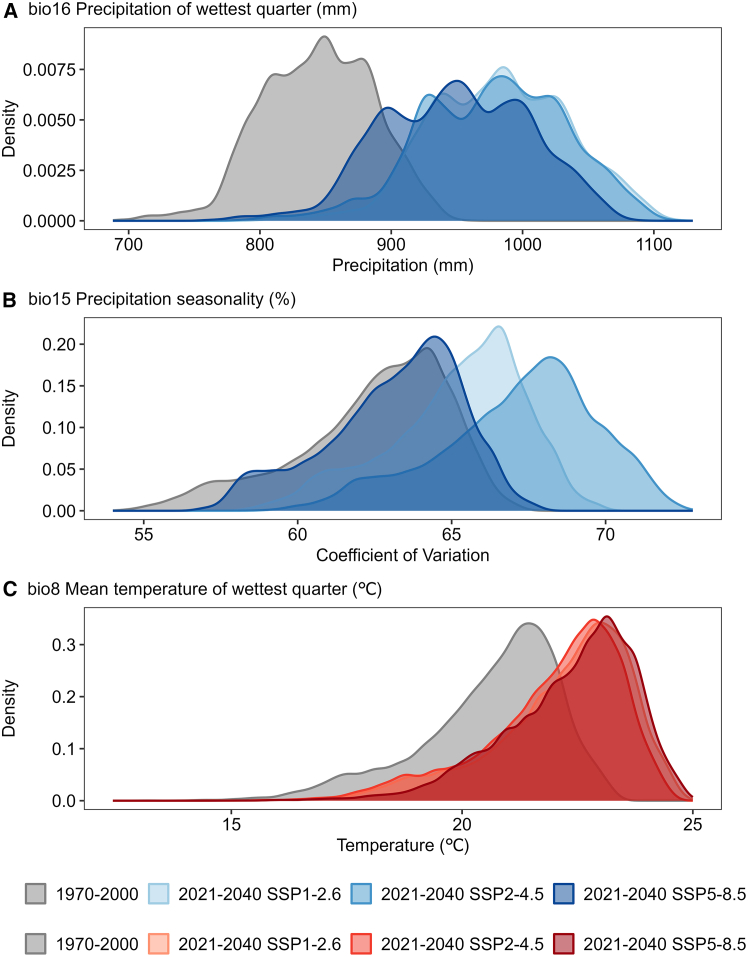
Environmental envelope drift analysis showing the shifts in probability density functions for core bioclimatic variables (A) bio16, precipitation of wettest quarter (mm); (B) bio15, precipitation seasonality (%); (C) bio8, mean temperature of wettest quarter (°C). The shifts in probability density functions compare the historical period (1970–2000) with future scenarios (SSP1-2.6, SSP2-4.5, and SSP5-8.5) for the near-term period (2021–2040).

Environmental envelope shift analysis based on probability density function displacement provides a mechanistic explanation for the widespread negative MESS values (Figure 10). Comparing key climatic variable distributions during the baseline period with those under three SSP scenarios for 2021–2040 reveals clear systematic shifts in core bioclimatic variables. For precipitation-related predictors, the probability density curve of bio16 shifts markedly rightward under future scenarios, indicating precipitation levels will frequently exceed historical upper bounds. Bio15 also exhibits a clear rightward shift, suggesting intensified intra-annual rainfall unevenness. Among temperature-related predictors, bio8 shows the most pronounced displacement, with future distributions almost entirely departing from the core region of the historical environmental envelope. This collective shift beyond the historical environmental envelope directly results in negative similarity scores in the MESS calculation, objectively demonstrating that future climate conditions are expected to exceed historical limits, necessitating model extrapolation under genuinely novel environmental conditions.

**Figure 11:**
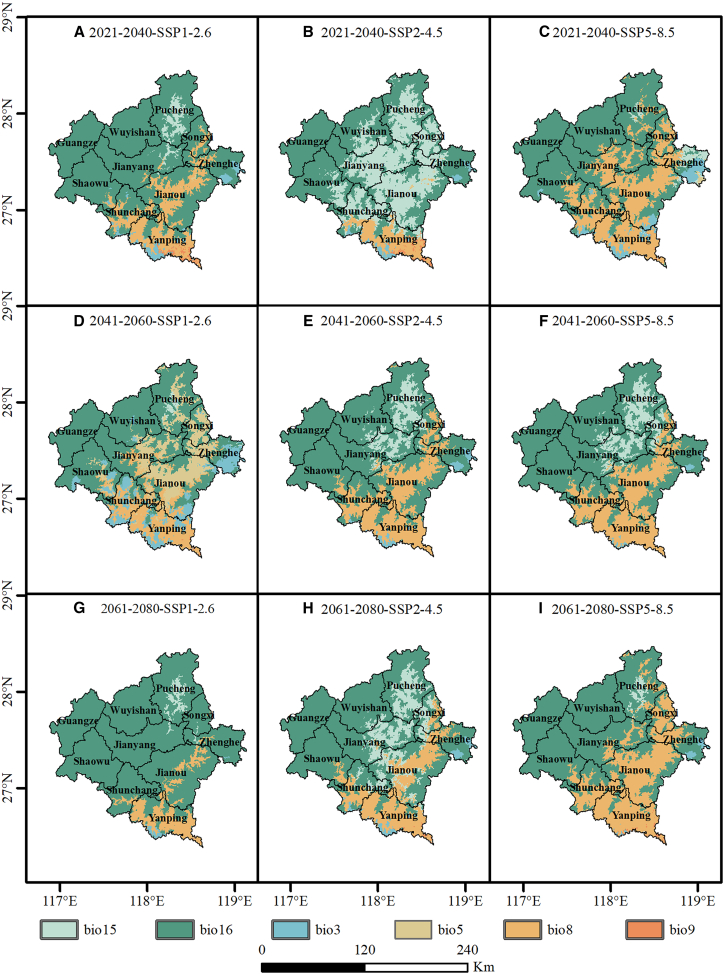
Spatial distribution of the MoD under future climate scenarios (A–C) 2021–2040 projections under SSP1-2.6, SSP2-4.5, and SSP5-8.5, respectively; (D–F) 2041–2060 projections under the three scenarios; (G–I) 2061–2080 projections under the three scenarios. The scale bar indicates 0–240 km.

Further examination via MoD analysis reveals distinct geographic differentiation in the environmental drivers responsible for high extrapolation risk across Nanping (Figure 11). In northern Nanping and surrounding Wuyishan areas, environmental novelty is primarily driven by precipitation-related variables, specifically bio16 and bio15. Conversely, in central and southern Nanping, bio3, and bio8 emerge as the principal drivers of environmental uncertainty. This spatial pattern indicates that increasing precipitation extremity and sustained warming during the wettest quarter represent the key forces driving climatic regime shifts in the region. Although this systematic climatic drift introduces extrapolation risk into model projections—implying that the projected expansion of suitable areas should be interpreted as a probabilistic outcome with inherent uncertainty—it also suggests that ongoing global warming may create unprecedented climatic resources facilitating potential tea cultivation introduction into higher latitude and broader regions. By identifying these dominant novelty drivers, the study provides a clearer mechanistic understanding of the environmental forces underlying projected distributional expansion, thereby offering a forward-looking risk framework for strategic planning of tea production under an increasingly uncertain climate future.

## Discussion

### Remote sensing-supported climatic suitability prediction

The integration of Gaofen-6 (GF-6) imagery with U-Net-based land cover classification to generate species occurrence records demonstrates significant innovation and application potential. We developed a standardized, transferable workflow that integrates high-resolution multispectral remote sensing with deep learning classification to automate the derivation of distribution maps and occurrence records from imagery. This approach substantially expands sample coverage while enhancing consistency and comparability^47^; systematic randomized sampling within the study area yielded 609 high-quality occurrence records. The MaxEnt model constructed with these records and 15 environmental predictors validates the efficacy and reliability of these data source for distribution modeling. Compared to traditional records primarily sourced from biodiversity networks, herbaria, literature, and field surveys, the remote sensing and deep learning methodology offers superior temporal efficiency, cost-effectiveness, and spatial resolution.^34^^,^^59^ Furthermore, contiguous cultivation patches derived from classification better reflect actual planting configurations, preserving agricultural morphology while reducing spatial biases from sparse point sampling. This enhancement provides MaxEnt with spatially complete inputs, thereby improving the characterization and interpretation of the realized niche.^60^ The 2 m resolution of GF-6, along with its red-edge band, significantly enhances the discrimination of spectrally similar evergreen vegetation.^61^ This capability demonstrates increased sensitivity in identifying linear, small-scale, or phenologically ambiguous tea plantations. Additionally, random sampling based on comprehensive distribution maps not only increases the sample size—thereby boosting model robustness and mitigating small-sample bias—but also optimizes spatial uniformity, effectively reducing errors induced by sampling bias.^62^^,^^63^

Within this integrated framework, the propagation of uncertainty from U-Net classification to MaxEnt predictions must be carefully addressed, as misclassification errors in remote sensing recognition may introduce spatial noise into the presence records. To mitigate this challenge, we implemented several constraints. First, the inherent high classification accuracy of the U-Net model ensures high-fidelity input data at the source. Second, the significant disparity in spatial resolution between the datasets serves as a critical scale buffer that dampens error propagation. Specifically, the U-Net-derived tea distribution maps possess a 2 m resolution, whereas the environmental predictors are at a 1 km resolution. This transition from fine to coarse scales acts as a physical filter; because each 1 km^2^ model grid cell aggregates a massive number of 2 m sub-pixels, fine-scale misclassification noise is effectively smoothed and neutralized during spatial aggregation or systematic random sampling.^64^ Furthermore, spatial thinning was applied to the presence data to eliminate environmental outliers, while the MaxEnt algorithm’s inherent robustness to moderate presence-point noise was further bolstered by optimizing regularization parameters via the ENMeval package. This calibration ensures that the model produces stable, conservative predictions rather than overfitting to potential noise.^65^^,^^66^ The high degree of spatial congruence between our predicted current high-suitability zones and actual production areas confirms that this strategy—combining high-resolution identification with moderate-scale modeling—successfully constrains error propagation within an acceptable range. In summary, the workflow based on GF-6 imagery and U-Net classification establishes a reproducible, high-precision pipeline, providing a robust operational foundation for spatial planning, cultivar introduction, and climate adaptation management in tea-producing regions.

### Environmental drivers of tea suitability

Through a synthesis of existing research, we preliminarily identified 45 environmental factors that influence the distribution of *Camellia sinensis*. These factors encompass bioclimatic variables, multi-depth soil attributes, topographic, and land-use types dimensions. Our analyses, employing Pearson’s correlation, variance inflation factor (VIF) metrics and jackknife tests, confirmed that bioclimatic variables predominantly affect the distribution, surpassing the influence of edaphic and topographic factors.^67^ In particular, the precipitation of the wettest quarter and precipitation seasonality collectively accounted for over 93% of the contribution to this distribution.

Precipitation serves not only as the fundamental water source for tea plants but also as a pivotal environmental signal modulating their phenological cycles. Our results demonstrate that the precipitation of the wettest quarter and precipitation seasonality exhibit exceptionally high contribution rates, profoundly reflecting the dependence of Nanping’s tea-growing regions on East Asian Monsoon patterns. The precipitation of the wettest quarter defines the productivity ceiling during peak harvest periods. Optimal rainfall maintains high relative humidity and minimizes leaf transpiration, effectively extending the tenderness period of the foliage—a physiological trait essential for elite cultivars such as Wuyi Rock Tea, which require specific leaf maturity profiles.^68^^,^^69^ However, the threshold response curves of bio16 suggest that excessive precipitation triggers multi-faceted adverse effects. On one hand, soil pore saturation leads to root hypoxia, restricting aerobic respiration and inhibiting the uptake of essential mineral nutrients, notably an045, while simultaneously exacerbating soil erosion in montane tea plantations.^70^^,^^71^ On the other hand, while high-humidity environments facilitate biomass accumulation, they significantly alter field microclimates and elevate the infection risk of fungal pathogens, such as tea anthracnose, thereby necessitating a biological trade-off between yield and quality.^57^ From a phenological perspective, precipitation seasonality governs the cyclic transition between dormancy, budding, and flushing by precisely regulating the temporal dynamics of wet-dry oscillations.^68^^,^^72^ During the pre-monsoon phase, the progressive increase in rainfall alleviates soil water stress and functions as a critical physiological trigger that induces the transition from winter dormancy to spring budding.^73^^,^^74^^,^^75^ Subsequently, the synergy between monsoonal moisture supply and rising temperatures creates a hydro-thermal synchronization effect; this enhances cellular turgor pressure to facilitate shoot expansion, increasing leaf elongation rates by over 50% during the monsoon season.^15^ This synchronized response provides the physiological foundation for the formation of multiple harvestable flush cycles.^13^ Furthermore, precipitation heterogeneity—represented by bio15—exerts a profound evolutionary regulatory effect on secondary metabolism. The transient, mild water stress occurring during the transition from the dry to the wet season can induce the accumulation of abscisic acid and activate corresponding signaling pathways.^67^^,^^76^^,^^77^ This physiological response does not merely optimize the biosynthetic equilibrium of secondary metabolites, such as catechins and aroma precursors; it also enhances the sensory quality of the tea through a subsequent burst of growth during the rapid expansion phase.^13^^,^^15^

Despite their lower contributions to distribution, topographic, edaphic factors, and land-use types hold significant practical importance. First, the analysis of land-use types reveals distinct landscape preferences for tea tree introduction. Model results indicate that SAV is the sole landscape category classified as highly suitable, followed by WSA, GRA, and ENF. From an ecophysiological perspective, this preference reflects the adaptation of high-quality tea cultivars to understory environments or forest-tea symbiotic systems. Moderate forest shading functions as a physical filter that attenuates direct solar radiation and increases the proportion of diffused light, thereby mitigating high-light-induced oxidative stress and facilitating the accumulation of amino acids—particularly theanine—in tea leaves.^78^^,^^79^ Furthermore, the classification of URB as an unsuitable class aligns with empirical cultivation logic and underscores the functional exclusivity of land-use variables in spatial planning. The inclusion of this variable filters out interference from non-productive zones such as urban centers and water bodies while characterizing the anthropogenic reshaping of natural environments. This landscape constraint ensures that the introduction zones predicted by the MaxEnt model possess practical feasibility rather than mere theoretical climatic suitability. Second, topographic factors influence the site conditions of tea plantations primarily by modulating hydro-dynamic properties.^57^^,^^80^ Within the complex montane terrain of Nanping, moderate slopes utilize gravity-driven flow to effectively prevent root hypoxia induced by waterlogging.^81^^,^^82^ This enhances the air exchange rate in the topsoil, thereby boosting root vitality.^83^ Such a physical drainage mechanism is critical for maintaining the physiological and metabolic equilibrium of tea trees amid the high precipitation of the wettest quarter, effectively bypassing root rot and nutrient-uptake barriers associated with anaerobic environments.^70^ Finally, soil physicochemical properties constitute the biochemical foundation of tea growth. As tea is a typically acidophilic and aluminum-tolerant crop, an acidic soil environment is a physiological prerequisite for maintaining normal nutrient absorption.^84^^,^^85^ In this study, the impact of AN045 is particularly vital. As a fundamental precursor for the synthesis of chlorophyll, proteins, and key flavor compounds, a sufficient supply of available nitrogen significantly enhances photosynthetic rates and delays leaf senescence, ultimately improving the umami and freshness of the tea.^86^^,^^87^^,^^88^ Moreover, higher soil clay content provides complex pore structures that not only facilitate moisture retention but also stimulate the activity of enzymes involved in nutrient cycling, thereby enhancing soil microbial diversity and the long-term stability of soil fertility.^89^

In conclusion, the threshold responses of bio15 and bio16 observed in this study align closely with established physiological and ecological mechanisms. However, it must be clarified that correlations derived from SDMs do not constitute direct causal evidence. To formally substantiate these inferences and quantify ecological effect sizes, future research must integrate long-term local phenological observations with *in-situ* physiological and biochemical assays, thereby bridging the gap between spatial patterns and mechanistic causality.^13^^,^^71^

### Tea climatic suitability under climate change

Utilizing three representative concentration pathways and ensemble global climate models, this study systematically projected the spatiotemporal evolution of tea cultivation suitability in Nanping from 2021 to 2080. Overall, under future climate scenarios, a majority of Nanping exhibits robust cultivation potential, with the Wuyishan core production zone maintaining its dominant status. Notably, climate change is facilitating the transition of previously unsuitable regions into viable habitats, providing a scientific basis for the large-scale southward migration and introduction of elite Wuyi tea cultivars.

Compared to the baseline period, the area of highly suitable habitats in Nanping is projected to undergo non-linear expansion by the 2040s, surging from 341.12 km^2^ to 22.12 × 10^3^ km^2^. Under baseline conditions, high suitability is strictly confined to the northern mountainous areas centered around the Wuyishan range due to winter low-temperature constraints or insufficient heat accumulation.^90^^,^^91^ As temperatures rise in the near- to mid-term, the cold limitation in south-central Nanping and low-altitude river valleys is effectively dismantled, aligning their climatic profiles with those of the Wuyishan core zone. This finding is logically complementary to the research of Tesfay et al.^71^ and the global synthesis by Jayasinghe and Kumar.^13^ While global tea suitability boundaries generally shift toward higher altitudes or latitudes to escape heat stress, in subtropical monsoon marginal zones like Nanping, initial moderate warming functions as a bioclimatic catalyst.^54^ Consequently, Nanping is poised to enter an expansive climatic window for the introduction of premium tea varieties over the coming decades.

Cross-regional comparative analysis reveals that Nanping possesses exceptional climate resilience. In contrast to low-latitude or plain production areas—such as regions in Sri Lanka and India that are already facing large-scale degradation of low-altitude tea plantations due to extreme thermal stress^17^—Nanping’s pronounced altitudinal gradient provides a critical buffer. When long-term SSP5-8.5 scenarios induce risks of thermal overload in low-elevation areas, the northern high-altitude zones will leverage their altitudinal compensation effect to offset the negative impacts of rising temperatures. Furthermore, the suitability dynamics in Nanping mirror those observed in the mountainous regions of Southwest China,^91^ where complex topography constitutes microclimatic refugia. Conversely, tea-growing regions in Eastern China and the Jiangnan plains, which lack topographic buffering, face significantly higher risks of habitat contraction.^14^

However, comparative analysis also unveils potential crises under high-emission scenarios. The localized retreat of suitability observed in the late-century SSP5-8.5 scenario reflects mechanistic interference with tea phenology caused by high temperatures. Extreme thermal stress not only restricts the physical distribution of tea plants but also disrupts secondary metabolic equilibrium, leading to decreased amino acid content and imbalanced tea polyphenol ratios, which compromises the characteristic flavor profile of Wuyi Rock Tea.^15^ In Nanping, this risk manifests as phenological disorders induced by hydro-thermal desynchronization. Given the significant contributions of bio15 and bio16 to suitability, extreme long-term warming may trigger an abnormal advancement of the spring flushing period. This exposes tender buds to severe temperature fluctuations in early spring or results in photoinhibition caused by the coupling of high temperature and high humidity in summer.^72^^,^^92^ Therefore, despite the immense short-term potential for cultivar introduction, long-term industrial planning must rely on the dynamic adjustment of altitudinal gradients to ensure that tea cultivation remains within its optimal climate envelope.

### Model consistency and reliability of suitability projections

Predictive uncertainty in species distribution modeling under future climate change primarily stems from algorithmic selection and shifts in climatic backgrounds. To address this issue, we employed the RF algorithm to cross-validate the MaxEnt results, providing an independent assessment of the identified suitability patterns. Although the RF model performs well, its TSS value was lower than both the MaxEnt counterpart and its own AUC score. This discrepancy can be largely attributed to the sensitivity of TSS to prediction threshold selection.^93^^,^^94^ Specifically, the MTSS threshold derived from RF was substantially higher than that of MaxEnt. While this improves specificity, it may exclude marginally suitable habitats, thereby reducing the overall TSS. Nevertheless, the high CBI suggests that RF maintains good calibration across the suitability gradient.^51^^,^^95^ Despite fundamental algorithmic differences, both the models exhibited strong spatial agreement in identifying the core high-suitability areas centered in Wuyishan City. This cross-algorithm consistency indicates that the spatial configuration of tea suitability in Nanping is more likely governed by dominant environmental drivers rather than biases specific to a single modeling approach. Such convergence among models based on different mathematical principles increases confidence in the robustness of the spatial patterns and supports the ecological plausibility of the projected suitability framework.^96^^,^^97^

To assess the reliability of projected suitable area expansion between 2021 and 2080, we applied MESS analysis to quantify uncertainty arising from spatial and temporal extrapolation. The results revealed pronounced environmental novelty across northern Nanping and the Wuyishan region, where MESS values were generally below −10. Environmental envelope shift analysis elucidates these negative values. Comparison of probability density functions between the baseline period and future climate scenarios showed a clear rightward shift in key variables, including bio16, bio15, and bio8. This displacement suggests future climatic conditions may fall outside the historical environmental envelope observed during 1970–2000. Consequently, strongly negative MESS values indicate that projections occur in novel environmental conditions lacking modern analogues, thereby increasing inherent extrapolation risks.^98^

From a physiological perspective, although projected shifts in bioclimatic variables entail statistical extrapolation risk, their direction is broadly consistent with the ecological preferences of tea plants. As detailed in section “environmental drivers of tea suitability,” adequate precipitation and suitable thermal conditions are the key factors constraining tea productivity. Environmental envelope shift analysis indicates a clear rightward displacement of these variables, suggesting that northern Nanping may experience a more abundant and synchronous supply of water and heat resources in the future. Previous studies have similarly shown that climate warming is a major driver of the northward and upslope expansion of tea cultivation.^14^^,^^54^^,^^91^ Therefore, although projected bio16 and bio8 values fall outside the historical range and yield negative MESS scores, the direction of change remains ecologically plausible for tea cultivation. In addition, changes in bio15 suggest a potential increase in precipitation seasonality, which, within Nanping’s subtropical monsoon climate, may still remain within the physiological tolerance and adaptive capacity of tea plants.^13^^,^^14^

### Limitations of the study

Despite improvements in model precision achieved through high-resolution remote sensing and spatial de-biasing, several limitations remain. First, because the model was calibrated on samples from a localized area, it faces the risk of niche truncation, whereby local occurrences may only represent a subset of the tea plant’s full climatic tolerance. This may introduce predictive bias when the model is extrapolated to broader geographic scales.^99^ Second, under extreme high-emission scenarios such as SSP5-8.5, future conditions may involve novel climates, namely environmental states that lack contemporary analogues within the current sampling range. Extrapolation into such unprecedented conditions remains a major source of uncertainty in species distribution modeling.^100^ Furthermore, tea plantations are highly anthropogenic systems whose spatial expansion is also influenced by non-climatic constraints such as land-use policies, economic incentives, and infrastructure. Consequently, projections based solely on bioclimatic variables may overlook these dynamic socioeconomic barriers.^13^^,^^54^ Future research should therefore incorporate nested model architectures or multi-source socioeconomic indicators to better quantify uncertainty during spatial transfer and provide a more robust scientific basis for regional planning and policy decisions.

## Resource availability

### Lead contact

Requests for further information and resources should be directed to and will be fulfilled by the lead contact, Xiaochen Zhu (xiaochen.zhu@nuist.edu.cn).

### Materials availability

This study did not generate new unique materials.

### Data and code availability

• All data and code used in this study are publicly available. The Gaofen-6 (GF-6) multispectral imagery was provided by the China Center for Resources Satellite Data and Application (Database: CRESDA: https://data.cresda.cn/). Bioclimatic variables were sourced from WorldClim (Database: WorldClim: https://worldclim.org/), and soil property datasets were obtained from the China National Tibetan Plateau Data Center (Database: TPDC: https://data.tpdc.ac.cn/). Topographic and land cover data were acquired from the Geospatial Data Cloud (Database: Geospatial Data Cloud: https://www.gscloud.cn/) and the MODIS Land Cover Type Product, respectively.
• The original U-Net code for tea plantation extraction and the generated occurrence records have been deposited at GitHub (Software: GitHub: https://github.com/verse-verse/U-Net).
• Any additional information required is available upon reasonable request to the lead contact.

## Acknowledgments

This work was supported by the Jiangsu Province Industry–University–Research Cooperation Project (Research and Development of Weather Forecasting Business Service Platform; grant no. BY20230008) and the Postgraduate Research and Practice Innovation Program of Jiangsu Province (grant no. KYCX24_1475).

## Author contributions

Conceptualization, supervision, funding acquisition, and resources, X.Z.; methodology, formal analysis, writing – original draft, and visualization, S.W.; validation, H.D.; writing – review and editing, P.T. and S.L.

## Declaration of interests

The authors declare no competing interests.

## STAR★Methods

### Key resources table

**Table:** 

REAGENT or RESOURCE	SOURCE	IDENTIFIER
**Deposited data**		

Gaofen-6 Satellite Imagery	CRESDA	https://data.cresda.cn/
WorldClim 2	Fick and Hijmans^101^	https://www.worldclim.org/
Dataset of Soil Properties for Land Surface Modeling over China	Dai and Shangguan^102^	https://data.tpdc.ac.cn/
Basic soil property dataset of high-resolution China Soil Information Grids (2010–2018)	Liu and Zhang^103^	https://data.tpdc.ac.cn/
ALOS PALSAR DEM (12.5 m)	JAXA/ASF	https://asf.alaska.edu/data-sets/sar-data-sets/alos-palsar/
MODIS Land Cover Type Product	Friedl and Sulla-Menashe^104^	https://lpdaac.usgs.gov/

**Software and algorithms**		

U-Net for tea plantation extraction	This paper	https://github.com/verse-verse/U-Net
MaxEnt v.3.4.4	American Museum of Natural History	https://biodiversityinformatics.amnh.org/
ArcGIS v.10.8	Esri	https://www.esri.com/
ENVI v.5.6	NV5 Geospatial	https://www.nv5geospatial.com/
Python v.3.8	Python Software Foundation	https://www.python.org/
R v.4.4.2	R Core Team	https://www.r-project.org/

### Experimental model and study participant details

This study does not use experimental models typical in the life sciences. The analysis is based on remote sensing data and environmental variables to assess tea plantation suitability. No specific biological strains or live plant subjects were used for laboratory experimentation.

### Method details

#### Remote sensing preprocessing and U-Net-based extraction of tea plantations

This research was conducted in Nanping City within the northern region of Fujian Province, China (26°30′–28°20′N, 117°00′–119°25′E), where the complex mountainous terrain and subtropical monsoon climate provide an optimal environmental envelope for high-quality tea cultivation (Figure S4).

To ensure consistency and reliability in model inputs, we utilized high-resolution Gaofen-6 (GF-6) multispectral imagery, provided by the China Center for Resources Satellite Data and Application (https://data.cresda.cn/#/home), to map the distribution of tea plantations in Wuyishan City. As China’s first high-resolution optical satellite designed for precision agriculture, GF-6 is equipped with a wide-field multispectral camera and a red-edge sensor, which, due to its high agility, is particularly suitable for crop classification and growth monitoring.^105^^,^^106^ Given the significant influence of tea phenology on spectral responses—such as the reflectance differences observed between the spring bud emergence and summer maturation phases^33^^,^^49^—we selected two 2-meter resolution scenes: 17 April 2021 (with less than 10% cloud cover) and 7 December 2021 (cloud-free), aligned with Wuyishan City’s tea phenological cycle. These datasets comprised RGB and infrared bands. The primary identification was conducted using the April imagery, while the December scene was employed to supplement areas affected by clouds, thereby facilitating the construction of a comprehensive tea plantation distribution dataset. Image preprocessing was conducted using ENVI 5.6, which included radiometric calibration, atmospheric correction, orthorectification, mosaicking, and cropping. A total of 2,961 sample points from tea plantations were generated through visual interpretation of contemporaneous Google Earth imagery.

To extract tea plantations in Wuyishan City from GF-6 imagery, we employed a U-Net convolutional neural network. The model processes four-band GF-6 imagery (blue, green, red, near-infrared) as input, producing binary classifications for tea and non-tea. As illustrated in Figure S5, the encoder consists of a four-stage hierarchy of stacked DoubleConv blocks, each comprising two 3 × 3 convolutional layers followed by Batch Normalization and ReLU activation. The channel depth progressively doubles from 64 to 512. Spatial downsampling is achieved through 2 × 2 max pooling between blocks. At the bottleneck, the feature channels expand to 1024 dimensions. The decoder features a symmetric design: transposed convolution upsampling is followed by skip-connection fusion with same-scale encoder features, culminating in feature refinement via DoubleConv blocks. The output layer generates class logits through 1 × 1 convolution, with pixel-wise category probabilities computed using Softmax activation.

For dataset construction, GF-6 imagery was partitioned into 256 × 256 pixel patches that were precisely co-registered with manually annotated binary masks. All patches were randomly divided into training and validation sets at an 80%:20% ratio. Model training was conducted over 100 epochs using the Adam optimizer and cross-entropy loss, while quad-metric monitoring was performed on both training and validation sets. Qualitative diagnostic plots, generated every 10 epochs, included RGB imagery, reference masks, and prediction masks to assess spatial consistency and boundary precision. Additionally, model weights were periodically saved to track the evolution of performance.

A sliding-window inference approach, utilizing a window size of 256 × 256 pixels and a stride of 256 pixels, was employed to generate a comprehensive map of Wuyishan City’s tea plantations. The trained model was applied to the entire GF-6 scene, with predictions from overlapping regions fused by retaining the maximum probability of tea plantation classification for each pixel. The resulting fused probability map was binarized using predetermined confidence thresholds, ultimately producing a georeferenced distribution map in GeoTIFF format. This probability-based mosaic fusion mechanism effectively minimized stitching artifacts while maintaining memory efficiency, thereby allowing for flexible adjustments in the trade-off between omission and commission errors.

#### Environmental variable selection and climate projections

Forty-five environmental variables across four dimensions—bioclimatic, edaphic, topographic and land cover (Table S1)—were selected based on existing research.^107^^,^^108^^,^^109^^,^^110^^,^^111^^,^^112^^,^^113^

Of these, nineteen bioclimatic variables were obtained from Global Climate Database (https://worldclim.org/), covering 30-arc-second resolution data for the 1970–2000 and three future periods (2021–2040, 2041–2060, 2061–2080). Future climate projections were generated using an equal-weight multi-model ensemble to reduce dependence on any single model. Three CMIP6 global climate models—BCC-CSM2-MR, MRI-ESM2-0, and EC-Earth3-Veg—were selected based on their structural independence and demonstrated skill in East Asian climate simulations. The models were chosen to capture complementary strengths, including representation of regional climate features, high skill in simulating East Asian monsoon precipitation, and incorporation of dynamic vegetation processes.^114^^,^^115^^,^^116^^,^^117^ Ensemble projections were derived by computing the arithmetic mean across the three model outputs for each variable and scenario. Projections correspond to three socio-economic forcing pathways (SSP1-2.6, SSP2-4.5, and SSP5-8.5), representing low, intermediate, and high emission trajectories, respectively. Previous evaluations indicate that these models reproduce historical temperature and precipitation patterns over southeastern China with acceptable skill. The dataset from 1970 to 2000 was utilized for model training and validation, while future scenarios projected climatic suitability.

Twenty-one edaphic variables were consolidated from two datasets archived at the China National Tibetan Plateau Data Center (https://data.tpdc.ac.cn/home): Dataset of Soil Properties for Land Surface Modeling over China^102^ provided alkali-hydrolyzable nitrogen, available phosphorus, and available potassium at 0–45 cm depth, while the Basic Soil Property Dataset of High-Resolution China Soil Information Grids^103^ supplied pH, total nitrogen, total phosphorus, total potassium, clay fraction, sand/silt/clay contents, and textural classes across multiple soil depths.

Topographic data were derived from a 12.5 m resolution DEM obtained via the Geospatial Data Cloud (https://asf.alaska.edu/data-sets/sar-data-sets/alos-palsar/). Land use data were derived from the MODIS Land Cover Type Product, classified according to the International Geosphere-Biosphere Program scheme. This dataset provides global land cover information at 500 m resolution, categorized into 17 distinct classes including various forest types, shrublands, savannas, grasslands, croplands, urban areas, and water bodies.^104^

All variables were uniformly clipped to Nanping City in ArcGIS 10.8, resampled to 30-arc-second resolution, reprojected to WGS 1984, and exported in ASCII format for use in MaxEnt modeling.

#### Modeling climatic suitability with MaxEnt

Occurrence records of *Camellia sinensis* were obtained from Wuyishan City, a representative tea-growing region in northwestern Nanping City, renowned for its Wuyi Rock Tea. This area is characterized by a warm-humid subtropical monsoon climate, featuring distinct seasons, abundant sunshine, and ample rainfall. The region is dominated by mountainous and hilly topography, with elevations ranging from 4 m to 2,149 m. These heterogeneous landscapes generate diverse microclimates that collectively create optimal conditions for tea cultivation.^118^^,^^119^ A total of 1,000 sample points were randomly generated from the U-Net-derived tea plantation distribution map. To reduce spatial redundancy, an environmental grid-based strategy was employed: bioclimatic grids at a resolution of 30 s served as spatial units, retaining only one representative point per unit, resulting in 609 final sample points (Figure S6). This method ensured spatial independence among samples and effectively mitigated model errors induced by spatial autocorrelation.

Given that spatial autocorrelation among environmental variables can introduce noise from highly correlated predictors and lead to model overfitting, the removal of redundant variables is critical for enhancing model transferability and ecological interpretability.^120^^,^^121^^,^^122^ The variable selection process was executed through a rigorous, multi-stage screening strategy to minimize multicollinearity while maximizing model parsimony. First, Pearson’s correlation analysis was employed to quantify pairwise relationships among all candidate predictors, identifying high correlations with a threshold of |r|>0.8 (Figure S7). Simultaneously, an initial MaxEnt model with 10 bootstrap replicates (training: test = 75%:25%) was run to establish the baseline percent contribution of each variable. Subsequently, a Variance Inflation Factor (VIF) analysis was conducted to further assess multicollinearity. To prevent the exclusion of ecologically significant predictors, we implemented a stepwise selection procedure: for variables exhibiting high VIF values (>10) or strong pairwise correlation, the predictor demonstrating a higher contribution rate in the initial model was prioritized for retention.^123^^,^^124^ A final diagnostic confirmed that all retained predictors possessed VIF values <10, ensuring statistical independence. This workflow yielded 15 core environmental predictors (an045, ap045, bio3, bio5, bio8, bio9, bio15, bio16, cly1530, slt1530, cf.1530, lu, pH515, slo, and tn515) for the final MaxEnt modeling.

In this study, MaxEnt (version 3.4.4) was employed to assess the climatic suitability and introduction potential for *Camellia sinensis* in Nanping City. To balance model complexity with generalization capability and mitigate overfitting, we conducted a rigorous hyperparameter tuning process using the ENMeval package in R.^125^ We performed a systematic grid search across eight Regularization Multipliers (RM: 0.5–4.0, step 0.5) and six Feature Class (FC) combinations (L, LQ, H, LQH, LQHP, and LQHPT).^125^^,^^126^^,^^127^ The optimal model configuration was selected based on a hierarchical set of criteria: (1) the model with the lowest Akaike Information Criterion corrected for small sample sizes was prioritized to ensure parsimony; (2) the 10% training omission rate was evaluated against the expected threshold of 0.1 to confirm prediction reliability; and (3) the difference between training and testing AUC values was minimized to maximize generalization performance.^125^^,^^128^ The optimized model was then applied to generate ensemble projections based on three GCMs, with all other settings consistent with standard protocols.

#### Model projection reliability assessment

In this study, we employed the Random Forest (RF) algorithm as an independent, third-party validator to further cross-validate MaxEnt’s spatial predictions and to ensure the high robustness of identified potential tea introduction areas. In SDMs, MaxEnt estimates the ecological niche from presence records and background samples, whereas RF—an ensemble of decision trees—makes no parametric assumptions and is well suited to capture complex nonlinear relationships and higher-order interactions among environmental predictors.^35^^,^^129^ If two algorithms with fundamentally different mathematical foundations produce highly consistent high-suitability spatial patterns, the ecological credibility of the predictions is substantially strengthened and algorithm-specific biases are mitigated.^130^

To ensure comparability between models, the RF was fitted using the same 15 selected environmental predictor variables as MaxEnt. In the data-building stage, in addition to the available high-quality presence records, we randomly generated 10,000 background points within Nanping to adequately sample the region’s available environmental envelope. Because RF can be prone to overfitting, we performed a systematic grid search to optimize key hyperparameters and balance model complexity against spatial generalizability. The search ranges were set as follows: number of trees (ntree) 70–1500, number of variables tried at each split (mtry) 1–10, and minimum node size (nodesize) 1–20. Based on cross-validation, the optimal combination was ntree = 70, mtry = 1, and nodesize = 1. This configuration constrained model complexity while maximizing spatial generalization, thereby improving stability without sacrificing predictive accuracy.

Given the severe class imbalance between presence records and background points, which can bias classifiers toward the majority class, we adopted a one-to-one bootstrap downsampling strategy. The RF was trained independently across 10 repeated iterations; in each iteration we randomly sampled, without replacement, a set of background points equal in number to the presence records as pseudo-absences to create a class-balanced training dataset, while the remaining background points were held out as an independent test set. This approach reduced bias from class imbalance and, by averaging across multiple iterations, decreased uncertainty introduced by random sampling, enhancing the robustness of model evaluation. After each bootstrap iteration we quantified RF performance using the evaluation metrics described above. We then averaged metrics across all 10 iterations and cross-compared the spatial projections with the MaxEnt baseline-period suitability map to assess the scientific validity and robustness of projected tea introduction-area expansions under future climate scenarios.

Although the cross-validation described above ensured the reliability of the modeling algorithms, projecting species distribution models into future climate scenarios inevitably involves spatial and temporal extrapolation beyond the range of historical observations, thereby introducing additional and quantifiable predictive uncertainty. To explicitly characterize this uncertainty, we applied a Multivariate Environmental Similarity Surface (MESS) analysis—commonly referred to as a novelty-layer analysis—to evaluate where and to what extent future climatic conditions deviate from the environmental envelope sampled during model training. Specifically, this approach quantifies environmental similarity at each grid cell by comparing multivariate combinations of projected climate predictors with the reference environmental space defined for the baseline period, thereby identifying regions where future climatic conditions fall outside the range represented in the training data.^131^^,^^132^ To focus the assessment on the climatic dimensions most relevant to tea distribution, the reference envelope was constructed from the multidimensional climatic space occupied by 609 spatially rarefied Camellia sinensis occurrence records and restricted to six primary bioclimatic predictors (bio3, bio5, bio8, bio9, bio15, and bio16). By comparing the ranges and multivariate positions of these core predictors between projection and baseline periods, MESS identifies which variables contribute most to environmental novelty and thereby supplies objective, geostatistical evidence to interpret modeled suitability changes.

### Quantification and statistical analysis

The performance of the U-Net model for tea plantation extraction was quantified using four standard metrics, including Accuracy, Precision, Recall, and F1-score. Accuracy represents the proportion of correctly classified pixels over the total number of pixels, while Precision and Recall respectively measure the reliability and completeness of the identified tea plantation class. The F1-score was used as a balanced indicator integrating both Precision and Recall. The mathematical formulations of these metrics are provided in Equations 1, 2, 3, and 4, where true positives, true negatives, false positives, and false negatives denote correctly and incorrectly classified tea and non-tea pixels.

(Equation 1) $$Accuracy=\frac{TP+TN}{TP+TN+FP+FN}$$

(Equation 2) $$Precision=\frac{TP+TN}{TP+FP}$$

(Equation 3) $$Recall=\frac{TP}{TP+FN}$$

(Equation 4) $$F1score=\frac{2\times Precision\times Recall}{Precision+Recall}$$

Building upon the evaluation of remote sensing classification accuracy, the predictive performance of the species distribution models was further assessed using a multi-metric framework. This framework incorporated the Area Under the Receiver Operating Characteristic Curve (AUC), the True Skill Statistic (TSS), and the Continuous Boyce Index (CBI) to capture different aspects of model performance. AUC was used to assess the model’s discriminatory ability independent of threshold selection. TSS quantified classification accuracy by accounting for both omission and commission errors, with values greater than 0.6 indicating reliable model performance. The CBI, specifically designed for presence-only data, evaluated the consistency between predicted suitability and observed occurrences, where values approaching 1 indicate strong model calibration across the suitability gradient.

Based on the continuous suitability outputs generated by the species distribution models, threshold-based classification was subsequently applied to enable spatial interpretation. Binary suitability maps were derived using the Maximum Training Sensitivity plus Specificity (MTSS) threshold, which was determined by maximizing the sum of sensitivity and specificity on the training dataset. This threshold converted continuous suitability outputs into binary presence–absence predictions, thereby optimizing the trade-off between omission and commission errors. To further refine spatial interpretation, continuous suitability values were stratified into ordinal classes. The MTSS value was defined as the lower bound of suitable habitat, and values exceeding this threshold were partitioned into equal intervals to delineate moderate, medium, and high suitability levels, ensuring consistency between threshold-based model evaluation and spatial classification.

Given that model projections under future climate scenarios inherently involve extrapolation beyond the observed environmental space, uncertainty was explicitly quantified using Multivariate Environmental Similarity Surface (MESS) analysis. Based on MESS scores, the study area was categorized into three levels of extrapolation risk. Areas with MESS values greater than 0 were considered reliable interpolation zones, where environmental conditions fall within the range of the training data. Areas with MESS values between −10 and 0 were defined as moderate-risk zones, indicating limited extrapolation beyond the historical environmental envelope. Areas with MESS values below −10 were classified as high-risk extrapolation zones, where projections occur under novel environmental conditions and are therefore associated with increased uncertainty.
