# Supplementary material for: MaxEnt with remote sensing for tea plantation suitability under climate change
Source: iScience. 2026 Apr 27;29(6):115887. doi: 10.1016/j.isci.2026.115887 (PMC13231076; doi:10.1016/j.isci.2026.115887)
Supplement: Document S1. Figures S1–S7 and Table S1 [file mmc1.pdf]

iScience, Volume *29*

Supplemental information

MaxEnt with remote sensing for tea plantation suitability under climate change

Shijie Wu, Pengfei Tian, Xiaochen Zhu, Hengxin Dong, and Shiwen Liu

Table S1. Environmental variables are used in this paper to model the cultivation distribution of *Camellia sinensis*.

Index	Name	Mean	Unit
Bioclimatic variables	bio1	Annual mean temperature	℃
	bio2	Mean diurnal range	℃
	bio3	Isothermality (bio2/bio7) (* 100)	%
	bio4	Temperature seasonality (standard deviation × 100)	-
	bio5	Max temperature of warmest month	℃
	bio6	Min temperature of coldest month	℃
	bio7	Temperature annual range (bio5-bio6)	℃
	bio8	Mean temperature of wettest quarter	℃
	bio9	Mean temperature of driest quarter	℃
	bio10	Mean temperature of warmest quarter	℃
	bio11	Mean temperature of coldest quarter	℃
	bio12	Annual precipitation	mm
	bio13	Precipitation of wettest month	mm
	bio14	Precipitation of driest month	mm
	bio15	Precipitation seasonality (Coefficient of variation)	%
	bio16	Precipitation of wettest quarter	mm
	bio17	Precipitation of driest quarter	mm
	bio18	Precipitation of warmest quarter	mm
	bio19	Precipitation of coldest quarter	mm
Edaphic variables	an045	Alkali-hydrolyzable nitrogen (0–45 cm soil depth)	$g \cdot kg^{-1}$
	ap045	Available phosphorus (0–45 cm soil depth)	$g \cdot kg^{-1}$
	ak045	Available potassium (0–45 cm soil depth)	$g \cdot kg^{-1}$
	som045	Soil organic matter (0–45 cm soil depth)	%
	pH515	Potential of hydrogen (5–15 cm soil depth)	/
	pH1530	Potential of hydrogen (15–30 cm soil depth)	/
	tn515	Total nitrogen (5–15 cm soil depth)	$g \cdot kg^{-1}$
	tn1530	Total nitrogen (15–30 cm soil depth)	$g \cdot kg^{-1}$
	tp515	Total phosphorus (5–15 cm soil depth)	$g \cdot kg^{-1}$
	tp1530	Total phosphorus (15–30 cm soil depth)	$g \cdot kg^{-1}$
	tk515	Total potassium (5–15 cm soil depth)	$g \cdot kg^{-1}$
	tk1530	Total potassium (15–30 cm soil depth)	$g \cdot kg^{-1}$
	cf515	Clay fraction (5–15 cm soil depth)	% by volume
	cf1530	Clay fraction (15–30 cm soil depth)	% by volume
	snd515	Sand content (5–15 cm soil depth)	$g \cdot kg^{-1}$
	snd1530	Sand content (15–30 cm soil depth)	$g \cdot kg^{-1}$
	slt515	Silt content (5–15 cm soil depth)	$g \cdot kg^{-1}$
	slt1530	Silt content (15–30 cm soil depth)	$g \cdot kg^{-1}$

	cly515	Clay content (5–15 cm soil depth)	$g \cdot kg^{-1}$
	cly1530	Clay content (15–30 cm soil depth)	$g \cdot kg^{-1}$
	texcls515	Soil textural classes (5–15 cm soil depth)	1: Sa; 2: LoSa; 3:Si; 4:SaLo; 5:Lo; 6:SiLo; 7:SaClLo; 8: ClLo; 9: SiClLo; 10: SaCl; 11:SiCl; 12: Cl
	texcls1530	Soil textural classes (15–30 cm soil depth)	
Topographic variables	alt	Altitude	m
	asp	Aspect	/
	slo	Slope	°
Land use	lu	land use	1: ENF; 2: EBF; 3: DNF; 4: DBF; 5: MF; 6: CSH; 7: OSH; 8: WSA; 9: SAV; 10: GRA; 11: WET; 12: CRO; 13: URB; 14: CNV; 15: SNI; 16: BAR; 17: WAT

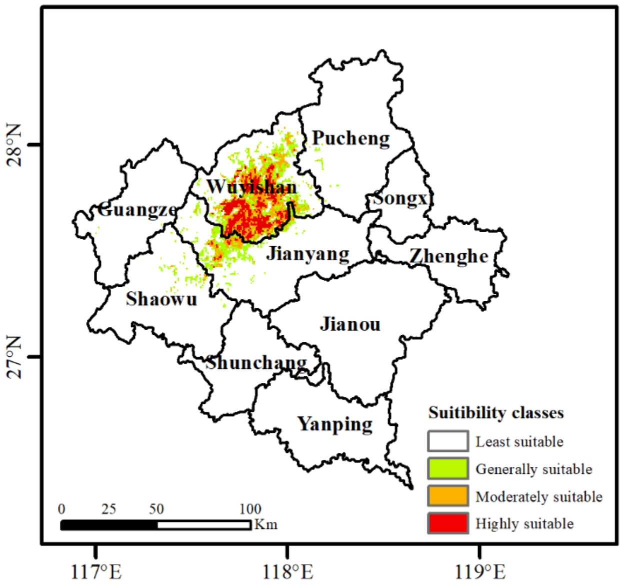

Fig S1. Current suitability classes map of Camellia sinensis based on the Random Forest model.

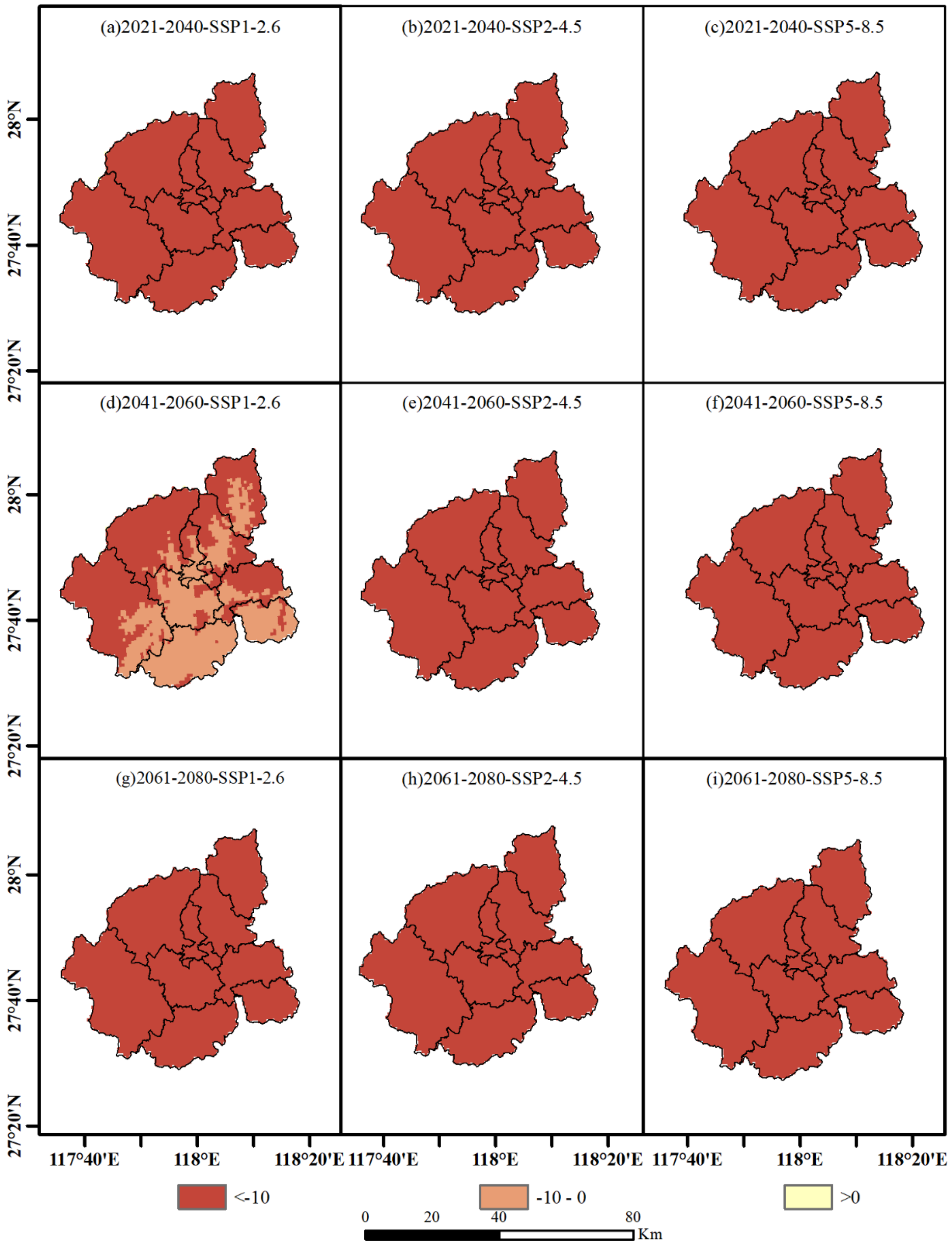

Fig S2. Spatial distribution of MESS analysis for Wuyishan City.

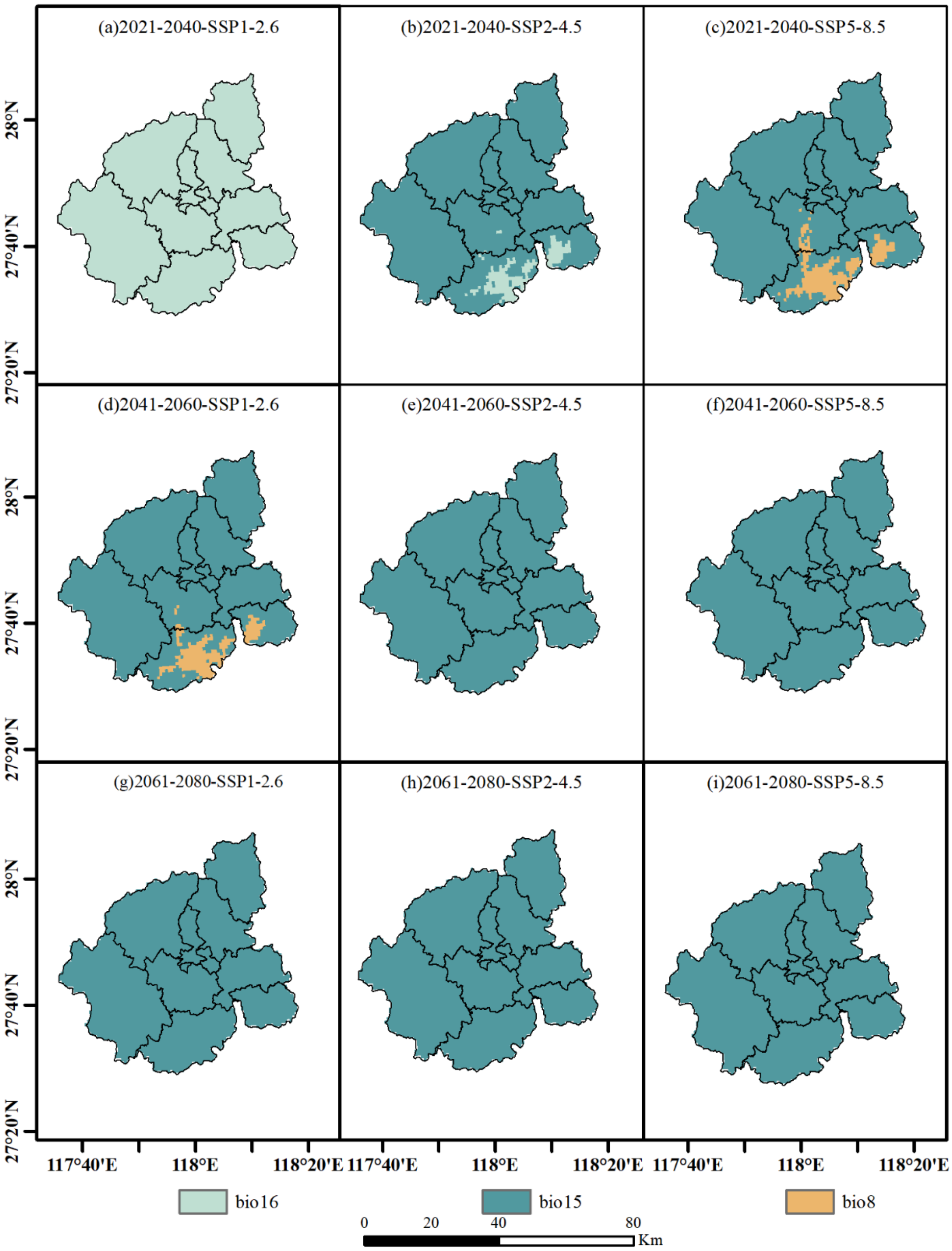

Fig S3. Spatial distribution of the MoD analysis for Wuyishan City

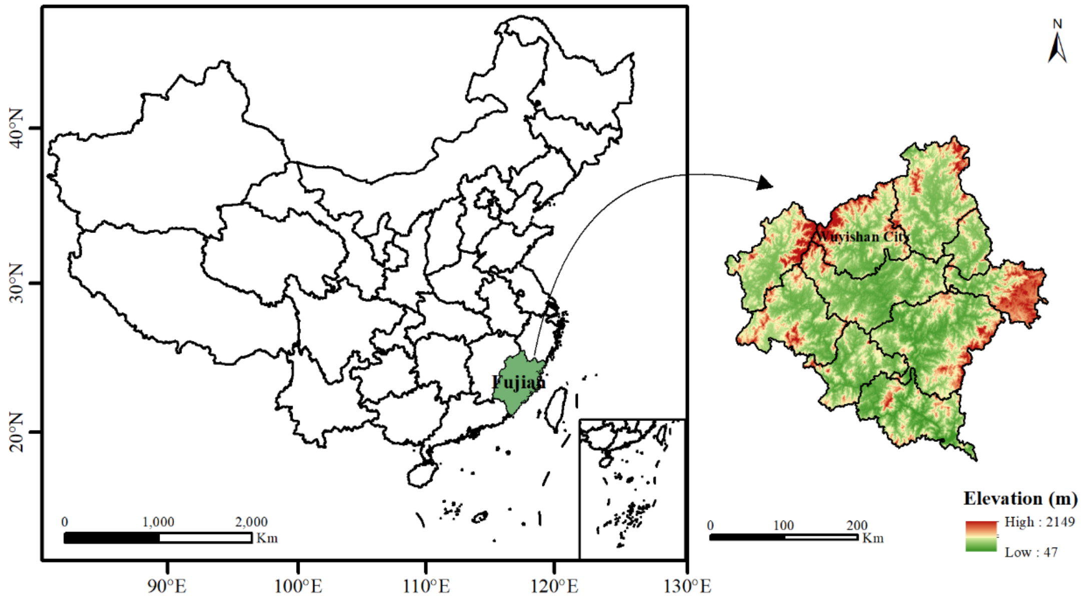

Figure S4. Geographical location of the study area.

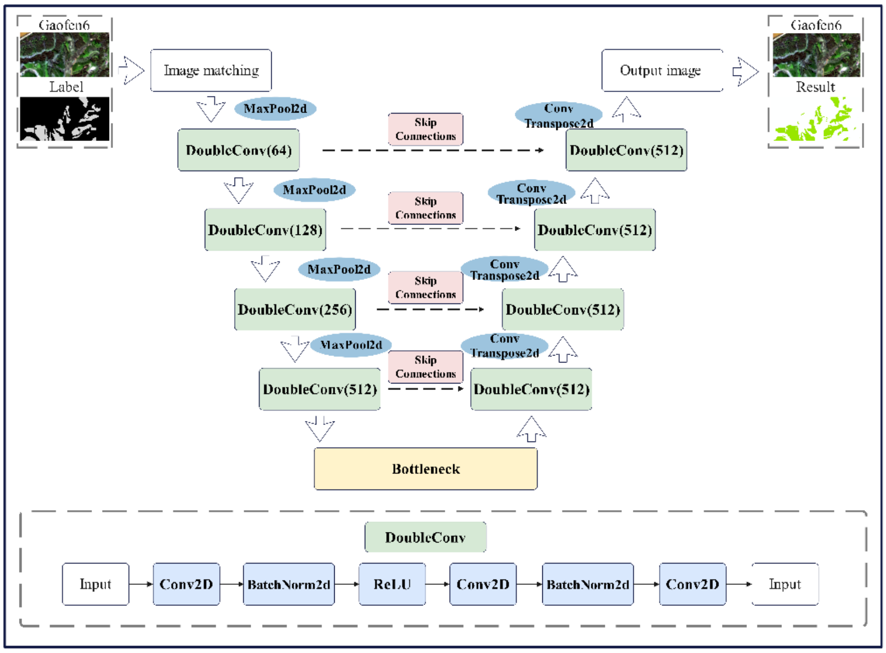

Figure S5. Schematic diagram of the U-Net model architecture.

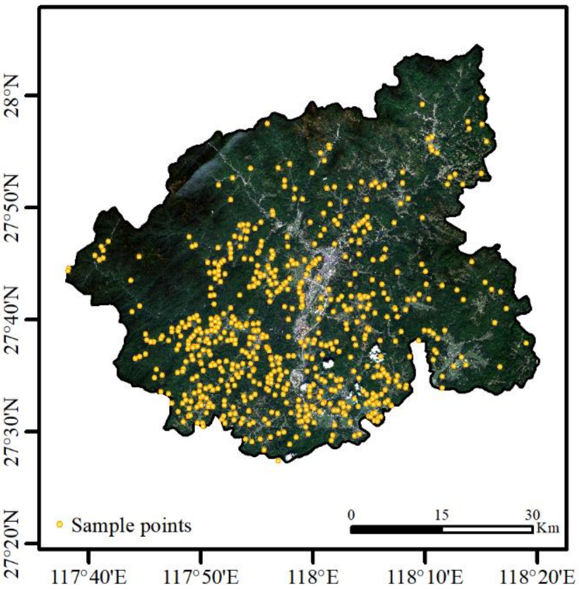

Figure S6. Corrected remote sensing imagery and sample point distribution in Wuyishan City (April 17, 2021).

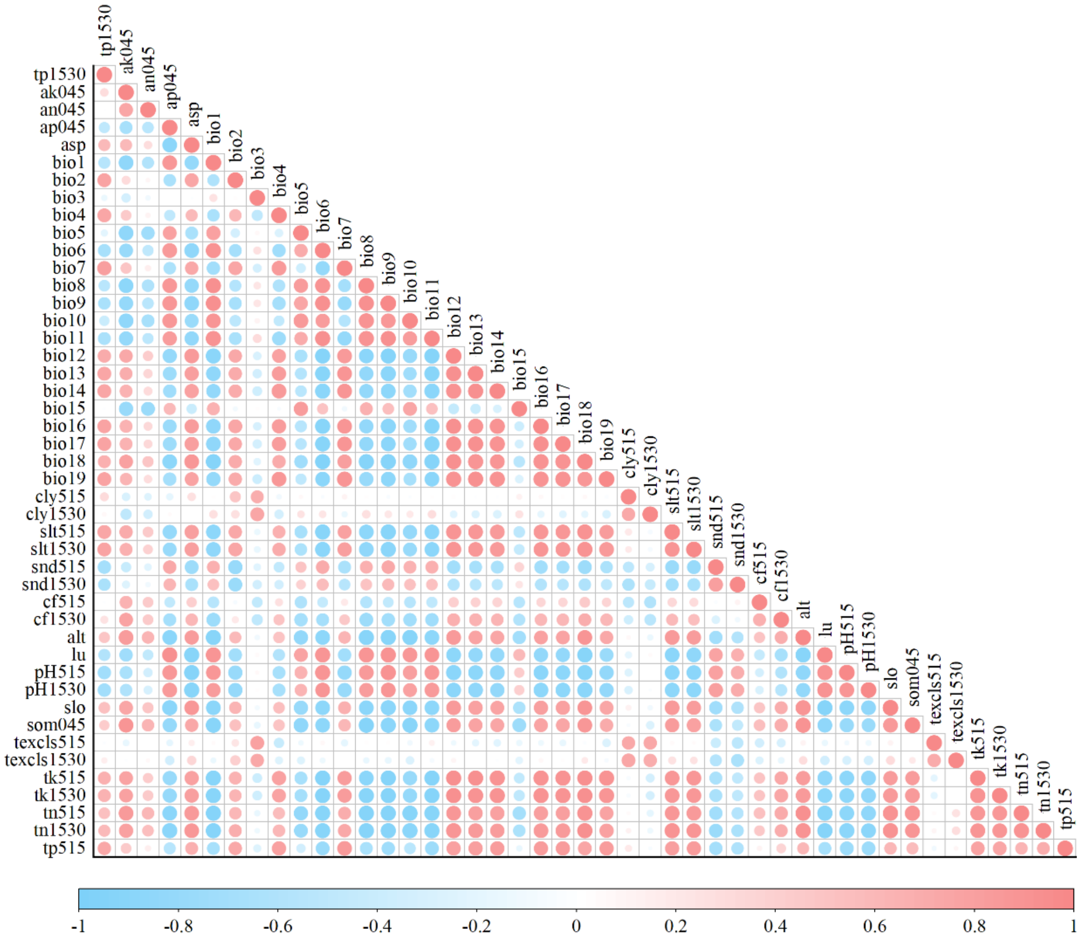

Figure S7. Correlation heat map of environmental variables.
